# Health Risks of Organophosphate Flame Retardants (OPFRs) in Facial Cosmetic Sponges via Dermal Exposure

**DOI:** 10.3390/molecules31071067

**Published:** 2026-03-24

**Authors:** Yang Yang, Yan Luo, Guiqin Liu, Jingfei Li, Xiangyong Meng, Cuicui Zheng, Zheng Zhang, Chun Yang, Jia Qiu, Hui Cao

**Affiliations:** 1 Zhejiang Institute of Quality Sciences, Hangzhou 310018, China; 2 Zhejiang Key Laboratory of Consumer Product Safety Research Under Provincial Market Supervision, Hangzhou 310018, China

**Keywords:** organophosphate flame retardants (OPFRs), cosmetic sponges, dermal exposure, migration, exposure risk

## Abstract

Organophosphate flame retardants (OPFRs) are widely used in consumer products and have attracted extensive attention due to their potential hazards. In this study, the concentration of OPFRs in cosmetic sponges, the migration of these compounds, and the assessment of dermal exposure risk are reported for the first time. Twelve OPFRs were detected in cosmetic sponges, with concentrations ranging from not detected (ND) to 9624 ng·g^−1^ and a total detection frequency (DF) of 75.58% (n = 86). A migration experiment was designed to evaluate the skin load of OPFRs from cosmetic sponges using the Strat-M^TM^ artificial membrane, and the reliability of the method was verified. The daily exposure of females (age: 11–40 years) to OPFRs through dermal contact with cosmetic sponges under different use conditions and for different age groups was assessed. The use of wet cosmetic sponges resulted in persistent and higher OPFRs exposure. Although the calculation of the hazard ratio indicated an acceptable health risk from OPFRs contained in cosmetic sponges, the toxicity results based on the L-929 cell line highlight that the potential toxicity risk caused by the migration of OPFRs from cosmetic sponges cannot be neglected.

## 1. Introduction

Organophosphate flame retardants (OPFRs), as the preferred alternative to traditional brominated flame retardants, have been widely used in the production of plastics, building materials, and electronic products [1,2,3,4]. However, OPFRs can be easily released into the environment through wear and tear, as they are often incorporated into materials via physical bonding [5]. As significant emerging contaminants, the potential health risks associated with OPFRs have garnered extensive attention worldwide. Humans can be exposed to OPFRs through multiple pathways, including inhalation of indoor air and dust, dietary intake, and dermal contact with consumer products [6,7,8]. OPFRs have been detected in human urine, breast milk, and serum, indicating widespread internal exposure [9,10]. Long-term exposure to OPFRs may adversely affect human health by impacting the reproductive, endocrine, and immune systems, as well as increasing the risk of cancer [11,12]. For instance, the Environmental Protection Agency (EPA) has determined that Tris(chloroethyl) phosphate (TCEP) has the potential to cause kidney cancer, damage the nervous system and kidneys, and impair fertility [13]. Triphenyl phosphate (TPhP) has been associated with endocrine disruption, showing anti-androgenic activity and altering hormone levels in animal studies [14,15]. Tributyl phosphate (TnBP) has been shown to cause neurotoxicity in occupational exposure settings [16].

Based on the Rapid Chemical Assessment Tool (QCAT), 97 OPFRs have been shown to be potentially toxic [17]. The Agency for Toxic Substances and Disease Registry (ATSDR) obtained the oral minimum risk level (MRL) for some OPFRs based on organ lesions observed in rats [18]. However, the EPA has not established an oral reference dose (RfD) or inhalation reference concentration (RfC) for OPFRs. Over the past few decades, OPFRs have been frequently detected in environmental substrates [19,20,21,22]. Notably, the presence of OPFRs in the human body has also been reported [10,23]. Therefore, the potential for human exposure to OPFRs from diverse sources and the associated health risks cannot be ignored.

Compared to environmental exposure, exposure to OPFRs in consumer products is often overlooked. Cosmetics and cosmetic tools have become essential products in daily life. However, compared with the standardized and strict management of chemical substances in cosmetics, consumer exposure to harmful substances in cosmetic tools has rarely been studied, such as beauty blenders and powder puffs, which are important tools used to evenly distribute cosmetics on the skin. OPFRs are widely used in foaming materials such as polyurethane, which is a common raw material for cosmetic sponges [24]. Frequent contact with cosmetic sponges may result in the transfer of harmful substances to facial skin [25,26,27,28]. For instance, Abdallah et al. investigated the absorption of chlorinated OPFRs into human skin using in vitro EPISKIN™ models, and concluded that OPFRs were adsorbed after 24 h of exposure [29]. Frederiksen et al. studied the permeability of skin to OPFRs in skin patches used in plastic surgery and found that OPFRs could accumulate on the skin [30]. Therefore, investigating the concentration of OPFRs in cosmetic sponges, their migration, and assessing the exposure risk are of great significance for protecting consumer health and the ecological environment.

Determining accurate migration volumes is important for reliable health risk assessment. The wipe paper method is commonly used for assessing dermal exposure to contaminants, where the amount recovered from the skin surface correlates strongly with internal exposure [3,27,31,32,33,34]. However, this method is not suitable for measuring contaminant transfer from cosmetic sponges, as these products are primarily used in a patting rather than wiping motion. This key difference in application mode necessitated the development of a new experimental approach that better simulates the actual contact between cosmetic sponges and skin. Strat-M^TM^ (Merck Millipore, Germany) is a synthetic membrane specifically designed to model human skin permeability. It comprises two layers of polyester sulfone containing synthetic lipids and a polyolefin supporting layer, with a lipid composition that mimics the stratum corneum of human skin [35,36,37]. Several validation studies have demonstrated its suitability as a substitute for human and animal skin in penetration research. Haq et al. reported a strong correlation between compound penetration through Strat-M^TM^ and human cadaveric skin [38], while Uchida et al. and Ameri et al. found that diffusion parameters of various chemicals in Strat-M^TM^ were comparable to those in excised human and hairless rat skin [39,40]. For cosmetic exposure scenarios specifically, Strat-M^TM^ offers several advantages. Unlike excised human or animal skin, which suffers from variability between donors and limited availability, Strat-M^TM^ provides consistent and reproducible barrier properties across batches—a critical factor for comparative migration studies. Its synthetic nature eliminates ethical concerns associated with human or animal tissue use. Furthermore, the membrane’s compatibility with cosmetic formulations and its ability to simulate the prolonged, repeated contact typical of cosmetic product use (such as the patting motion of cosmetic sponges) make it particularly well-suited for assessing dermal exposure to chemicals in cosmetic tools. These characteristics support its use as the migration medium in our study.

In this work, OPFRs in cosmetic sponges were analyzed using liquid chromatography–tandem mass spectrometry (LC-MS/MS). A migration experiment was designed to monitor the transfer of OPFRs from cosmetic sponges using the Strat-M™ membrane, and migration was measured under different conditions. The human health risk was assessed by determining the daily exposure dose and hazard quotient (HQ)—a metric used to characterize non-carcinogenic risk. Further analysis was carried out for different user groups, ages, and genders. The individual and cumulative health risk due to OPFRs in cosmetic sponges was comprehensively evaluated. This study provides additional information on the health risks associated with the frequent use of cosmetic sponges and is expected to help agencies properly regulate the use of OPFRs in cosmetic sponges.

## 2. Results and Discussion

### 2.1. OPFRs Concentrations in Cosmetic Sponges

The heatmap of OPFR concentrations, generated after normalizing the maximum content of each OPFR in 86 samples, is presented in Figure S1, where the red area indicates a higher relative concentration level. Preliminary observation suggested that the overall concentration of OPFRs in all samples was relatively low, with TPhP, TEP, and TiBP frequently detected. The DF and concentrations of the OPFRs in the cosmetic sponges are summarized in Table 1. Twelve OPFRs were identified in the 86 samples. Total OPFRs (ΣOPFRs) were quantified with a DF of 75.6% at concentrations ranging from ND to 9624 ng·g^−1^. Σaryl-OPFRs exhibited the highest DF of 59.3%, with a concentration range of ND to 7196 ng·g^−1^. Four aryl-OPFRs were identified, with TPhP (ND-7196 ng·g^−1^) showing the highest DF (45.4%). A total of six alkyl OPFRs (Σ alkyl-OPFRs) were detected, with a DF of 53.5% and concentrations ranging from ND to 9424 ng·g^−1^. TiBP exhibited the highest concentration, ranging from ND to 9399 ng·g^−1^. For the OPFRs containing a halogen group, TCEP (DF = 3.49%) and TCPP (DF = 10.5%) were detected at concentrations ranging from ND to <LOQ, and ND to 2386 ng·g^−1^, respectively. TiBP, TPhP, EHDPP, TPPO, and TCPP were the main substances of interest due to their high concentrations. The major OPFRs identified in cosmetic sponges—TiBP, TPhP, EHDPP, TPPO, and TCPP—are all compounds commonly used as flame retardants and plasticizers in polyurethane foam manufacturing [41]. For example, TCPP is the dominant flame retardant in flexible polyurethane foams due to its high efficiency and low cost [42]. TiBP and TPhP are frequently added as plasticizers to improve foam flexibility and durability [43,44]. TPPO may be present as a processing aid or residual compound from polymerization [45]. Since cosmetic sponges are primarily made from polyurethane, the presence of these OPFRs is consistent with their intentional addition during foam production.

**Figure 1:**
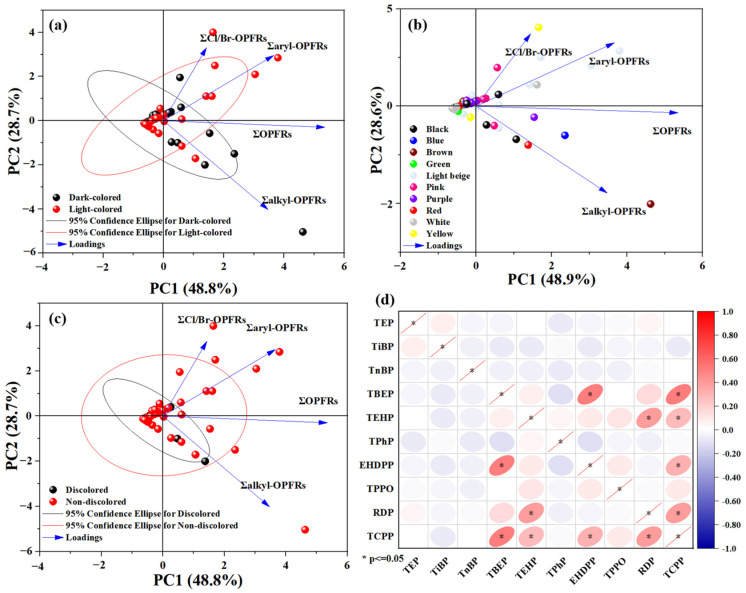
PCA of the measured OPFRs in dark-colored and light-colored cosmetic sponges (**a**), sponges with different colors (**b**), discolored and non-discolored samples (**c**); (**d**) heat map of the correlation between different OPFRs concentrations.

The distribution of OPFRs in cosmetic sponges was further analyzed based on color. The samples examined in this study were initially categorized into dark-colored and light-colored groups; detailed information is presented in Table S1. The results for the classified samples are summarized in Table S2. In the principal component analysis (PCA), the 95% confidence ellipses for the dark- and light-colored samples showed some overlaps, indicating that there is consistency between the two groups of data in dark and light colors (Figure 1a). The samples were further classified according to specific colors, and the results are compiled in Table S2. However, no significant difference in the concentrations of the OPFRs was observed for the samples of different colors according to PCA (Figure 1b). During acetonitrile extraction of the sample in the pretreatment step, some colored samples were decolorized (Table S1). Plausibly, the decolorized samples did not contain industrial additives such as color-fixing agents. The samples were subsequently divided into decolorized and non-decolorized categories, and the relationship between the decolorization phenomenon and the OPFRs was analyzed. No significant difference in the OPFRs concentrations was found for the non-decolorized and decolorized sponges, suggesting that the OPFRs present in the various colored materials were not significantly related to the decolorization (Table S3, Figure 1c). The correlations between the concentrations of the individual OPFRs in the makeup sponges were investigated (Figure 1d). The concentrations of TBEP, EHDPP, and TCPP were significantly positively correlated (*p* < 0.05), whereas the concentration of TEHP was significantly positively correlated with that of RDP (*p* < 0.05). This correlation suggests that these substances might originate from the same source in the industrial process of manufacturing cosmetic sponges. A positive correlation among several substances could indicate that the population is susceptible to simultaneous exposure to these OPFRs.

Currently, there are no reports on the occurrence of OPFRs in cosmetics or cosmetic tools and the associated risk. In a study of industrial products, Zhang et al. reported that the total concentration of OPFRs in toys collected from China ranged from 6.82 to 228,254 ng·g^−1^ [46]. Wu et al. found that the concentration of OPFRs in child seats varied from 1900 to 44,000,000 ng·g^−1^ [47]. Stubbings et al. detected TPhP at concentrations ranging from 410 to 9,900,000 ng·g^−1^ in children’s polyurethane foam (PUF) mats in the United States [48]. Gill et al. analyzed the content of flame retardants in upholstered furniture components and reported a range of 1160–49,800 mg·kg^−1^ [49]. In comparison to these products, the concentration of OPFRs in the cosmetic sponges analyzed in this study was significantly lower. When OPFRs were intentionally added to a product, their content typically constituted 5–15 wt.% of the polymer material [50]. However, the concentrations of OPFRs measured in the cosmetic sponges were evidently below this threshold. Furthermore, the median concentrations of various OPFRs in the cosmetic sponges analyzed herein were all below the LOQ, suggesting that these compounds were not conventional ingredients in the production of cosmetic sponges. Plausibly, OPFRs may originate from migration of plastic packaging, either through the use of recycled materials [51,52] or by absorption from the environment during production or storage [53].

The concentrations of OPFRs on the surface and in the inner core of the cosmetic sponges were analyzed to determine whether these compounds originated from external pollution. Based on the shape of the sponges (flat or spherical), five samples of powder puffs and beauty blenders were selected to analyze the content of OPFRs on the surface and within the inner core. The test results are presented in Table S4. The mean concentration of each substance was calculated, and a bar chart was created, as illustrated in Figure S2. For flat powder puffs, there was minimal difference between the concentrations of the OPFRs in the inner core and on the surface due to the thinner shape. For approximately spherical beauty blenders, the concentrations of TEP, TiBP, TEHP, TPPO, and TCEP on the surface and within the inner core were similar. However, the surface concentrations of TPhP (798 ng·g^−1^), EHDPP (424 ng·g^−1^), and TCPP (622 ng·g^−1^) were much higher than those of the inner core (251, 33.2, and 98.2 ng·g^−1^, respectively). The results suggest that TPhP, EHDPP, and TCPP in beauty blenders probably originated from migration from external packaging; thus, the concentration was high on the surface and low in the inner core. Although OPFRs in cosmetic sponges have been shown to be added unintentionally, the potential risks to consumers arising from exposure need to be further clarified.

### 2.2. Migration and Exposure to OPFRs

Before conducting a risk assessment for harmful substances in a product, it is essential to consider factors such as the characteristics of the products, users, and use scenarios. In view of the makeup application being predominantly associated with females, and considering the noticeable trend of younger demographics engaging in makeup application, this study primarily focuses on the exposure of females aged 11 to 40 years to OPFRs. The use of cosmetic sponges is generally categorized into two modes: one involves directly patting the dry sponge on the face, while the other entails wetting the sponge with water before use. Consequently, the amount of OPFRs migrating to the artificial membrane under both dry and wet conditions was tested. The wet sponge used for the test was obtained by fully absorbing deionized water and then squeezing out excess water with a tablet press (5 MPa). Two types of samples were selected, and the water content was calculated by gravimetric determination (Table S5 and Figure S3). The saturated water absorption of beauty eggs and powder puffs was approximately between 6.93 and 10.40 and 3.53–6.63 g·g^−1^, and the moisture content after squeezing was approximately between 1.29 and 1.97 and 0.77–1.59 g·g^−1^. The migration experiments were carried out with the samples having OPFR concentrations near the 95th percentile values to ensure the reliability of the data [46] (n = 9), and the results are presented in Table S6.

(1) Dry cosmetic sponges

**Figure 2:**
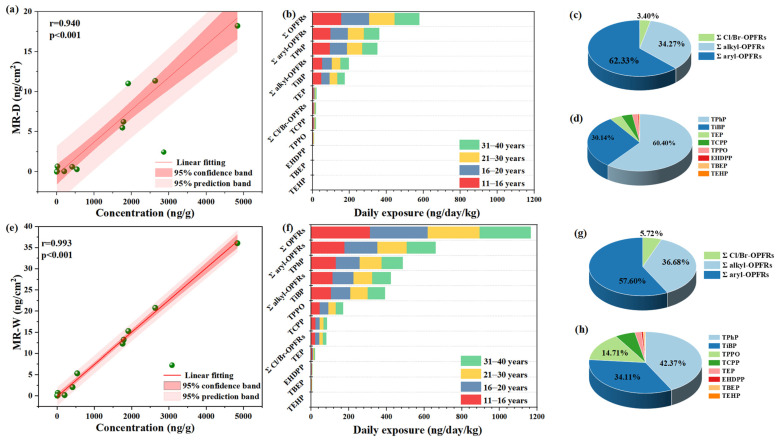
Correlation analysis between migration and concentration of OPFRs wiped from (**a**) dry and (**e**) wet cosmetic sponges; mean daily exposure (ng·kg^−1^·day^−1^) for female aged 11–40 years to OPFRs from (**b**) dry and (**f**) wet cosmetic sponge; exposure contribution of OPFRs under (**c**,**d**) dry and (**g**,**h**) wet conditions.

First, for all samples, a certain amount of OPFRs migrated to the simulated skin. The *C_area_* for ΣOPFRs in dry cosmetic sponges ranged from 10.4 to 34.0 ng·cm^−2^ (Table S6). As shown in Figure 2a, the *C_area_* for the OPFRs was significantly positively correlated with the concentration of OPFRs in the sponges (r = 0.940, *p* < 0.001). Thus, the migration determined using the simulated method is reliable. The skin exposure for females of different ages was calculated by Equation (1), and the results are displayed in Table S7. The daily dermal exposure to ΣOPFRs through dry makeup sponges in females aged 11 to 40 years ranged from 77.2 to 286 ng·kg^−1^·day^−1^. The mean exposure of females of different ages to ΣOPFRs and individual OPFRs across nine samples was calculated, and the results are presented in Table 2 and Figure 2b. The mean exposure of females aged 11–16, 16–21, 21–30, and 30–40 years to ΣOPFRs was 153, 143, 138, and 135 ng·kg^−1^·day^−1^, respectively. As the age increased, the daily exposure dose gradually decreased, attributed to the increase in body weight. Therefore, special attention should be paid to the exposure risks faced by the adolescent population using these cosmetic sponges. The contribution of various types of OPFRs to the overall exposure was further analyzed, and the results are presented in Figure 2c. Among the three categories of OPFRs, Σ aryl-OPFRs contributed significantly to exposure (62.33%), followed by Σ alkyl-OPFRs (34.27%) and Σ Cl/Br-OPFRs (3.40%). For individual OPFRs, TPhP contributed the most (60.40%, Figure 2d), with daily exposure levels ranging from 45.1 to 135 ng·kg^−1^·day^−1^ (Table S7). The mean values for exposure to TPhP across the four age groups were 95.6, 89.4, 85.7, and 84.2 ng·kg^−1^·day^−1^, respectively. TiBP also demonstrated a significant contribution (30.14%), with mean values for the four age groups of 46.4, 43.3, 41.5, and 40.8 ng·kg^−1^·day^−1^, respectively. Furthermore, the level of exposure to TEP, TCPP, TPPO, EHDPP, TBEP, and TEHP was lower due to less migration (Table S6, Figure 2b,d).

(2) Wet cosmetic sponges

The sponges were wetted with deionized water before conducting the migration test. Migration of the OPFRs at various concentration levels was detected in all samples, where the specific migration results are presented in Table S6. The C_area_ for ΣOPFRs tested in the wet sponges ranged from 15.5 to 64.7 ng·cm^−2^. Notably, migration from the wet sponges was generally higher than that from the dry sponges. This increased migration under wet conditions is likely due to water penetrating and swelling the polyurethane matrix, which facilitates OPFR diffusion to the surface [55], combined with the mechanical squeezing during application that further promotes release from the sponge. However, no significant correlation was observed between the water content and the migration amount. The C_area_ for OPFRs also exhibited a significant positive correlation with the concentration of OPFRs detected in the sponges (*r* = 0.993, *p* < 0.001) (Figure 2e). Similarly, the daily exposure dose of OPFRs for females aged 11–40 years was calculated based on migration from wet cosmetic sponges, which ranged from 114 to 545 ng·kg^−1^·day^−1^ (Table S8). The average daily exposure for ΣOPFRs and each OPFR in all samples is displayed in Table 2 and Table S8, Figure 2f. For the age groups of 11–16, 16–21, 21–30, and 30–40 years, the daily exposure to ΣOPFRs was 304, 284, 272, and 267 ng·kg^−1^·day^−1^, respectively, where the values are almost twice the exposure from dry sponges. The significantly higher exposure level is attributed to the greater migration of OPFRs from wet cosmetic sponges. As shown in Figure 2g, the contribution of Σ alkyl-OPFRs, Σ aryl-OPFRs, and Σ Cl/Br-OPFRs to the exposure from wet cosmetic sponges was 36.68%, 57.60%, and 5.72%, respectively. Furthermore, after the sponges were wetted, the contributions of TPPO and TCPP increased from 1.67% and 3.39% to 14.71% and 5.72%, respectively (Figure 2h), which might be related to the hydrophilicity associated with the relatively low log Kow values of TPPO (2.87) and TCPP (2.89) (Table S9). Overall, there were obvious differences in the migration of OPFRs based on the usage mode, where wet cosmetic sponges would result in a greater exposure risk.

Generally, cosmetic sponges can be reused many times; thus, the migration of OPFRs from wet cosmetic sponges used ten times was further evaluated to determine the exposure from repeated use. A sample with a high migration level (T-33) was selected for the test, and the amount of OPFRs that migrated with each use is shown in Figure S4. The main substances that migrated from this sample were TEP, TiBP, and TPhP. As shown in the figure, the amount of TEP and TiBP that migrated was relatively stable during ten consecutive uses, in the range of 0.62–1.29 and 2.85–5.34 ng·cm^−2^, respectively. The migration of TPhP ranged from 3.88 to 41.68 ng·cm^−2^, where the migration was highest for the first use. Thereafter, migration gradually decreased with repeated use. Analysis of the internal and external concentrations of the samples (Figure S2) in the sponge indicated that TPhP most likely originated from external contamination, resulting in higher surface concentrations and, therefore, higher migration during the first use. The results indicate greater exposure when the sponge is used for the first time. Although the migration gradually decreased with repeated use, migration still resulted in continuous exposure for users. Therefore, if a harmless cosmetic sponge is not strictly selected, exposure to OPFRs would be continuous. Chronic low-dose exposure to OPFRs has been associated with endocrine disruption, reproductive toxicity, and other adverse health outcomes in epidemiological and animal studies [56]. Therefore, while the *HQ* values calculated in this study suggest an acceptable risk under the current exposure scenarios based on single-use data, the cumulative effect of repeated exposure over years of regular use warrants further investigation. Future studies should consider longer-term repeated-use experiments and biomonitoring approaches to assess internal exposure levels in regular cosmetic sponge users.

For better comparison with relevant studies, reports on the dermal exposure of consumers to OPFRs from certain products are summarized in Table 3. For exposure from a single product source, Tang et al. analyzed the exposure of occupational workers at electronic waste dismantling sites to OPFRs from hand wipes, with a median exposure to OPFRs of 183 ng per hand wipe. Assessment of the exposure doses indicated that TPhP contributed the most (2.07 ng·kg^−1^·day^−1^) [27]. Zhang et al. determined migration (26.2 ± 46.7 ng·cm^−2^) by wiping the surface of toys, and conducted a dermal exposure assessment for children aged 3 months to 6 years (21.4 to 52.4 ng·kg^−1^·day^−1^) [46]. Similarly, Peng et al. reported that the exposure of children under 5 years to OPFRs via dermal contact with play pads was 24–29 ng·kg^−1^·day^−1^ [57]. Zhu et al. found that the average dermal exposure dose of OPFRs in baby clothes ranged from 0.92 to 1.13 ng·kg^−1^·day^−1^ [58]. In this study, the exposure level from cosmetic sponges was significantly higher than the dermal exposure associated with the aforementioned consumer products. In addition to the exposure from a specific product, environmental exposure associated with consumer products has also been reported [10,48,59,60]. For example, Fan et al. determined the exposure data for children in kindergarten by wiping their foreheads, toys, and desks, with dermal exposure calculated as 3710 ng·kg^−1^·day^−1^ [59]. Stubbings et al. assessed exposure to 16 OPFRs from dust and indoor air in childcare centers in Seattle, USA, and found OPFR concentrations of 4100–29,000 ng·g^−1^ and dermal exposures of 4890 ng·kg^−1^·day^−1^ [48]. However, in most studies, the environmental exposure levels tended to be even higher. Moreover, all exposure associated with consumer products contributes directly or indirectly to environmental exposure. Therefore, the health risks and environmental impact caused by consumer products such as cosmetic sponges should not be ignored.

### 2.3. Risk Assessment

The safety risk due to exposure to OPFRs from cosmetic sponges was evaluated by calculating the *HQ* values. The EPA considers a total *HQ* value of 1.0 to be an acceptable threshold, below which the hazard risk is acceptable. The *RfD* for the OPFRs, except TPPO, were obtained from literature reports, and the most unfavorable values were adopted [2,46,62]. The *RfD* of TEP, TPhP, TiBP, TCPP, EHDPP, TBEP, and TEHP is 125, 7, 100, 3.6, 0.6, 20, and 35 μg·kg^−1^·day^−1^, respectively.

**Figure 3:**
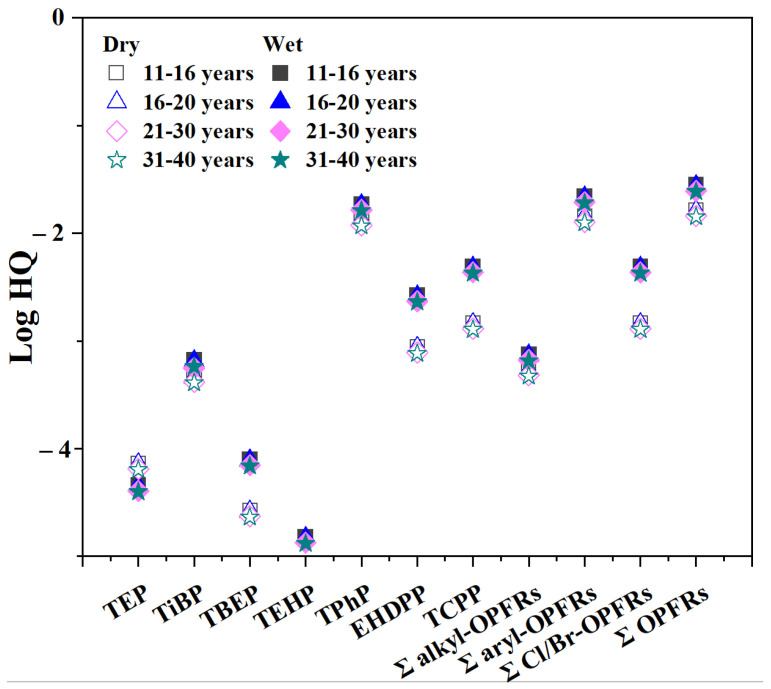
Log transformation of HQ of OPFRs by females in different age groups via dermal contact.

The *HQ* values for different users were calculated and are summarized in Tables S10 and S11. For adolescent and adult females, the HQ for all dry sponges ranged from 7.0 × 10^−3^ to 3.0 × 10^−2^ (Table S10), whereas the *HQ* for wet sponges ranged from 7.6 × 10^−3^ to 6.4 ×10^−2^ (Table S11). The mean value of Σ*HQ* for each age group is presented in Table 2. In this study, the *HQ* values for the use of cosmetic sponges across all age groups were below the non-carcinogenic risk threshold limits established by the EPA. This result suggests that exposed individuals are unlikely to experience adverse non-carcinogenic risks, as expected. The logarithmic plot of the transformation of the HQ values for the individual OPFRs across different age groups for all users is presented in Figure 3. The data at the top represent a higher risk, with TPhP identified as the substance requiring primary attention. Additionally, the figure presents a comparison of the differences in the risk due to dry and wet makeup sponges. The migration and risk associated with TEP and TEHP were not significantly influenced by the two usage regimes. However, the risk was higher for the remaining OPFRs when the cosmetic sponges were wet, especially for EHDPP and TCPP. A limitation of this study is that the findings are specific to the 11–40 age group and non-occupational users, and may not be applicable to other populations. In addition, several sources of uncertainty should be considered when interpreting our risk characterization. Exposure parameters represent maximum scenarios. The 100% absorption assumption likely overestimates actual uptake. Migration data from high-concentration samples may overestimate exposure from typical products. Collectively, these uncertainties suggest our calculated HQ values represent upper-bound estimates, and this conservative approach ensures potential health risks are not underestimated for a screening-level assessment.

### 2.4. Cytotoxicity Assessment

According to the migration obtained from the experiment, standard solutions of corresponding concentrations were prepared using water for cytotoxicity tests. The concentrations of TEP, TiBP, TBEP, TEHP, TPhP, EHDPP, TPPO, and TCPP were 0.008, 0.099, 0.005, 0.005, 0.088, 0.005, 0.273, and 0.063 μg·L^−1^, respectively. The 24 h cytotoxicity of the samples to the L-929 cell line was evaluated by MTT assay. Firstly, the morphology of the cells was observed. Before adding the sample, the cells underwent normal proliferation. At a sample concentration of 25%, slight growth inhibition and occasional cytolysis were observed. At sample concentrations of 50% and 75%, up to 70% of the cells were round or lysed, with over 50% growth inhibition. At 100% sample concentration, the cell layer was nearly destroyed. As shown in Table S12, dose-dependent behavior was observed during the exposure period. The cells had an acceptable survival rate (77.0%) at 25% sample concentration. However, at 100% sample concentration, the activity decreased to 3.4% after 24 h of exposure. Therefore, the exposure concentration of this sample has potential cytotoxicity to L-929 cells. Although the calculated *HQ* values were below 1, suggesting an acceptable risk under the assessed exposure scenarios, the observed cytotoxicity indicates that the compound has intrinsic bioactive potential. These combined findings highlight the importance of integrating both risk assessment and hazard identification approaches and suggest that continued monitoring and further studies with additional endpoints are warranted.

This study focused on the initial OPFR concentrations in cosmetic sponges and the amounts migrating to a simulated skin membrane—parameters that are essential for estimating dermal exposure and calculating health risks. This study has several limitations that should be acknowledged. First, the risk assessment was based solely on dermal exposure via cosmetic sponges, which likely represents a minor contribution to total daily OPFR intake compared with other significant pathways such as indoor dust ingestion, dietary intake, and inhalation. Therefore, the low HQ values estimated here do not imply negligible population-level risks from OPFRs. Second, the cytotoxicity experiments, while indicating intrinsic bioactive potential, were conducted in vitro and may not directly translate to in vivo effects. Future studies should integrate multi-pathway exposure assessments and additional toxicological endpoints to provide a more comprehensive risk profile. In addition, several limitations regarding sample representativeness should also be acknowledged. First, all samples were collected from online platforms in China, which may limit global generalizability due to regional differences in regulations and manufacturing practices. Second, detailed information on brands and material compositions was not available for some samples due to incomplete product labeling. Third, the sample size of 86, while reasonable for an exploratory study, may not capture the full diversity of cosmetic sponge products available. Future studies with broader geographic sampling would improve representativeness and enable cross-regulatory comparisons. Despite these limitations, this study offers important contributions by providing the first comprehensive assessment of OPFRs in cosmetic sponges and demonstrating that migration from wet sponges results in higher exposure levels. The findings underscore the importance of integrating both risk assessment and hazard identification approaches, and highlight the need for continued monitoring—particularly for sensitive populations such as adolescents who face higher exposure levels during critical stages of growth and development.

## 3. Materials and Methods

### 3.1. Chemical Reagents

The detailed information of the 25 target OPFRs is shown in Table 1 and Table S9. All the OPFRs were divided into three groups according to functionality, namely, alkyl-OPFRs, aryl-OPFRs, and halogenated (Cl/Br)-OPFRs. The Strat-M^TM^ membrane (diameter of 25 mm) was produced by Merck Millipore (Darmstadt, Germany). *N*-propyl ethylenediamine adsorbent (PSA) powder was obtained from Welchrom (Shanghai, China). Column chromatography silica gel (Si, 200–400 mesh) was purchased from Sigma-Aldrich (Steinheim, Germany).

### 3.2. Sample Preparation and Instrumental Analysis

#### 3.2.1. Sample Collection and Extraction Procedure

A total of 86 cosmetic sponges were collected from various online shopping platforms in China, including https://www.alibaba.com, https://www.taobao.com, and https://www.pinduoduo.com. Various retailers were evaluated to determine the cosmetic sponges with the highest sales. Detailed sample information, including reported material composition (e.g., polyurethane, latex) as obtained from product labels, is provided in Table S1. The cosmetic sponges were cut into pieces measuring less than 5 × 5 × 5 mm using scissors and stored in a clean, sealed bag prior to extraction. The OPFRs in the cosmetic sponges were extracted and analyzed using the method previously reported by our group [61]. The specific procedure is as follows: 0.2 g of the sample was placed into a 50 mL centrifuge tube and subjected to ultrasonic extraction for 30 min twice in succession with 10 mL of acetonitrile. The supernatants from the two extractions were combined, and the total volume was adjusted to 20 mL. Subsequently, 200 mg of Si and 200 mg of PSA were added to 2 mL of the extract, and the mixture was swirled for 1 min. The resulting solution was then filtered through a nylon filter membrane (0.22 μm) and transferred into a sample bottle for measurement [61].

#### 3.2.2. LC-MS/MS Analysis

OPFRs in the samples were detected using an LC-20AD liquid chromatograph (Shimadzu, Kyoto, Japan) and a Q-TRAP 5500 triple quadrupole–tandem mass spectrometer (AB SCIEX, Framingham, MA, USA) in positive electrospray ionization (ESI^+^) mode, employing multiple reaction monitoring (MRM). Mass spectrometry ion-pair information was obtained using the methods previously reported by our group (Table S13) [61]. A Phenomenex Kinete^TM^ C_18_ column (2.6 μm particle size, 3 mm × 150 mm, Los Angeles, CA, USA) was utilized with a flow rate of 0.4 mL·min^−1^ at 40 °C. The target OPFRs were separated by gradient elution with 0.1% formic acid in water (solvent A) and methanol (solvent B), using an injection volume of 2 μL. The gradient is summarized in Table S14.

### 3.3. Migration Test

A migration experiment was designed to observe the transfer of the OPFRs from cosmetic sponges to the human face and to quantify the skin load of these substances. The Strat-M^TM^ membrane was chosen to simulate human facial skin. The specific steps for determining the migration quantity are as follows:

(1) Simulation of migration scenario: The Strat-M^TM^ membrane was affixed to a flat operating table. A cosmetic sponge containing OPFRs was attached to a commercially available electric powder puff (rated power: 5 W, rated voltage: 5 V)—a cosmetic tool designed for foundation application. The switch of the device was activated to generate a specific slapping frequency on the artificial membrane, thereby simulating the process of dabbing the cosmetic sponge on the face.

(2) Migration experiment: The electric device was positioned above the artificial membrane, allowing it to continuously slap the surface of the artificial membrane for 30 s at a fixed frequency of approximately 3 times per second. The sponge was attached at a consistent position, and the distance to the membrane was calibrated prior to each experiment to maintain similar contact conditions. The diameter of the artificial membrane was 25 mm.

(3) Extraction of OPFRs from artificial membranes and analysis: After completing the migration experiment, the entire artificial membrane was cut into pieces (<5 × 5 mm) for extraction, and all pieces were transferred to a clean centrifuge tube with 1 mL of acetonitrile. The centrifuge tube was ultrasonically extracted for 30 min to fully extract the OPFRs from the artificial film. The resulting solution was filtered and analyzed using LC-MS/MS to determine the concentration of OPFRs.

The mass of OPFRs in the membrane was normalized to the area concentration (C_area_, ng·cm^−2^), which represents the concentration of OPFRs transferred from the cosmetic sponge to the skin. A clean Strat-M^TM^ membrane was tested without conducting the migration experiment to establish a blank control and consider background contamination.

### 3.4. Exposure and Risk Assessment

In light of the fact that cosmetic sponges are primarily used for dermal contact, a skin exposure model was employed to investigate the exposure to OPFRs. The daily dermal exposure (*E*, ng·kg^−1^·day^−1^) was estimated based on Equation (1) [63]:

(1) $$E=\frac{C_{area}\times CA\times AF\times t}{BW}$$

where *C_area_* (ng·cm^−2^) is the concentration of OPFRs transferred from the cosmetic sponge to the face, as determined from the migration test (refer to the steps in Section 2.3). *CA* denotes the contact area (cm^2^), which corresponds to the area of the user’s face. The facial area constitutes approximately 3% of the total surface area of human skin, as referenced in the *EPA Exposure Factors Handbook* and *The SCCS Notes of Guidance* [54,64]. AF indicates the absorption factor, regulatory guidance from agencies like the European Chemicals Agency, and National Medical Products Administration of China recommends using 100% absorption as a default value for initial screening when empirical data are lacking [65,66]; therefore, AF is defined as 100% in this study, *t* represents the daily occurrence frequency (times·day^−1^), and *BW* is the body weight of the individual (kg).

The non-carcinogenic health risk was assessed using the hazard quotient (*HQ*), defined as the ratio of the estimated daily dermal exposure dose (*E*) to the reference dose (*RfD*). The *HQ* value was calculated according to Equation (2) [67].

(2) $$HQ=\frac{E}{RfD}$$

*RfD* refers to the reference dose for daily exposure to OPFRs that does not result in significant harmful effects (measured in ng·kg^−1^·day^−1^). The *RfD* used in this calculation was obtained from literature reports for the OPFRs detected in this study and is presented in the Results and Discussion section. *HQ* < 1 indicates that exposure to the chemical does not pose a serious risk. Conversely, *HQ* > 1 suggests an increased potential for harmful effects.

### 3.5. In Vitro Cytotoxicity Test

Cell culture and treatment: L-929 cells were cultured in Minimum Essential Medium (MEM) medium containing 10% fetal bovine serum and antibiotics (penicillin 100 IU/mL, streptomycin 100 µg/mL) in an incubator (5% CO_2_, 37 °C, >90% humidity). The cells were digested with 0.25% trypsin (including EDTA) to prepare a single-cell suspension. The cell suspension was centrifuged (200× *g*, 3 min), the cells were re-dispersed in the medium, and the cell density was adjusted to 1 × 10^5^ cells·mL^−1^ of cell suspension. The cell suspension was inoculated into a 96-well culture plate with 100 µL per well and cultured in an incubator (5% CO_2_, 37 °C, >90% humidity) for 24 h.

Cytotoxicity evaluation: The cytotoxicity of the OPFRs with exposure concentration to L-929 cells was detected by 3-[4,5-dimethylthiazol-2-yl]-2,5-diphenyltetrasodium bromide (MTT) assay [46]. After the cells grew into a monolayer, the original culture medium was discarded, and 100 µL of the test sample solution with different concentrations (100%, 75%, 50%, 25%), blank control, positive control (100%), or negative control solution (100%) was added. The sample solutions with concentrations of 75%, 50%, and 25% were diluted with the 100% concentration sample solution and blank control extract (medium containing 10% fetal bovine serum). The samples were cultured at 37 °C with 5% CO_2_ for 24 h. Six parallels experiments were conducted for each group. After 24 h, the 96-well plates were removed for morphological observation of the cells. The original culture medium was discarded, 50 µL of MTT (1 mg·mL^−1^) was added to each well, the supernatant was discarded after 2 h, and 100 µL of isopropyl alcohol was added to each well for dissolution and crystallization. The absorbance was determined at 570 nm with 650 nm as the reference wavelength, and the cell survival rate (%) was calculated as follows:

(3) $$Cell\;survival\;rate=\frac{OD_{570e}}{OD_{570b}}\times 100$$

where *OD_570e_* is the average absorbance of the sample solution, and *OD_570b_* is the average absorbance of the blank control.

### 3.6. Quality Assurance and Quality Control

The OPFRs were quantified using external standard curves with six concentration levels (0.5–20 ng·mL^−1^). The regression coefficient (*r*) of all analytes was greater than 0.999. The accuracy of the method was verified through a matrix spike recovery test. The recoveries of the compounds in the cosmetic sponge samples ranged from 61.1% to 115%, and those in the artificial film samples ranged from 63.8% to 129% (Table 4). Laboratory procedural blanks were analyzed for every 10 samples to assess potential contamination. TEP was detected in the program blank with a low concentration of 1.1 ± 0.2 ng/mL; this value was subtracted from the concentration of the analytical sample. In the migration test, a clean Strat-M^TM^ membrane was tested without conducting the migration experiment to establish a blank control and consider background contamination. For every 10 samples, a midpoint calibration standard was injected to check the sensitivity drift of the instrument. Three parallel samples were set up, and the mean and standard deviation (SD) were calculated to ensure the reliability of the results. The limit of detection (LOD) and the limit of quantitation (LOQ) of the OPFRs were calculated from the mean concentration using a signal-to-noise ratio of 3 and 10, respectively. For OPFR concentrations below the LOQ, statistical analysis was conducted using LOQ/2 as a substitute. The normality of the data was assessed using the Shapiro–Wilk test (*p* > 0.05). Pearson correlation analysis was employed to examine the relationship between the migration volume and concentration of OPFRs in the cosmetic sponges.

## 4. Conclusions

Cosmetic sponges are frequently used in the makeup process, and the health risks due to the contact of chemicals with facial skin are often overlooked. In this study, the occurrence of OPFRs and the associated risk from dermal exposure due to the use of cosmetic sponges are reported for the first time. Twelve OPFRs with different detection frequencies and concentrations were found in 86 samples. Analysis of the concentration of OPFRs on the sample surface and within the inner core indicates that OPFRs in cosmetic sponges may originate from unintentional addition, such as the use of recycled materials, packaging, and environmental contamination. Migration was measured by using a Strat-M^TM^ membrane to simulate facial skin, and exposure and risk assessments were performed. This study revealed that OPFRs migrate from both dry and wet cosmetic sponges to facial skin. The amount of OPFRs that migrate from wet cosmetic sponges is higher, resulting in greater exposure levels and health risks. Although the *HQ* is below the risk threshold, cytotoxicity experiments highlighted the potential toxicity stemming from OPFRs in cosmetic sponges. Adolescents are in critical stages of growth and development, making them particularly sensitive and susceptible to harmful substances. Moreover, the widespread detection and prevalence of OPFRs in cosmetic sponges, along with their frequent use in daily life, make it necessary to pay attention to persistent health risks. Therefore, it is recommended that manufacturers establish stricter raw material controls to avoid OPFR-contaminated recycled plastics, and that regulatory bodies consider including cosmetic tools under existing chemical safety frameworks to establish specific OPFR limits.

## Figures and Tables

**Table 1:** Information, detection frequencies (DF, %), and concentrations (ng·g^−1^) of OPFRs in all samples (n = 86).

Types	Name	Analytes	DF (%)	Range (ng·g^−1^)
alkyl-OPFRs	Tris(methyl) phosphate	TMP	0.00	ND
	Tris(ethyl) phosphate	TEP	24.4	ND–285
	Tris(propyl) phosphate	TPrP	0.00	ND
	Tris(isopropyl) phosphate	TiPP	1.16	ND–<LOQ
	Tris(butyl) phosphate	TnBP	3.49	ND–871
	Tris(isobutyl) phosphate	TiBP	32.6	ND–9399
	Tris(hexyl) phosphate	THP	0.00	ND
	Tris(2-ethylhexyl) phosphate	TEHP	12.8	ND–54.2
	Tripentyl phosphate	TPEP	0.00	ND
	Tris(2-butoxyethyl) phosphate	TBEP	10.5	ND–101
aryl-OPFRs	Tris(phenyl) phosphate	TPhP	45.4	ND–7196
	Tris(4-isopropylphenyl) phosphate	TiPPP	0.00	ND
	Trixylyl phosphate	TXP	0.00	ND
	Triphenylphosphine oxide	TPPO	14.0	ND–3128
	2-Ethylhexyl diphenyl phosphate	EHDPP	16.3	ND–2057
	3-Methylphenyl diphenyl phosphate	MDPP	0.00	ND
	Tetraphenyl resorcinol bis (diphenylphosphate)	RDP	2.33	ND–153
	Bisphenol-A bis (diphenyl phosphate)	BDP	0.00	ND
	Tris-m-cresyl phosphate	TMCP	0.00	ND
	Tri-o-cresyl Phosphate	TOTP	0.00	ND
	tris(2,6-dimethoxyphenyl) phosphane	TDMPP	0.00	ND
Cl/Br-OPFRs	Tris(chloroethyl) phosphate	TCEP	3.49	ND–<LOQ
	Tris(chloropropyl) phosphate	TCPP	10.5	ND–2386
	Tris(2,3-dichloropropyl) phosphate	TDCP	0.00	ND
	Tris(2,3-dibromopropyl) phosphate	TDBPP	0.00	ND
/		Σ alkyl-OPFRs	53.5	ND–9424
/		Σ aryl-OPFRs	59.3	ND–7196
/		Σ Cl-Br-OPFRs	10.5	ND–2386
/		ΣOPFRs	75.6	ND–9624

Note: DF: detection frequency; ND: not detected.

**Table 2:** Input parameters for exposure assessment, daily exposure of ΣOPFRs and ΣHQ for OPFRs from cosmetic sponges at different ages and genders.

Age Group	BW ^a^ (kg)	SA ^b^ (m^2^)	CA (cm^2^)	EF (Times·Day^−1^)	Daily Exposure to ΣOPFRs (ng·kg^−1^day^−1^)		ΣHQ	
					Dry	Wet	Dry	Wet
11–16 years	55.9	1.57	477	1	153	304	1.6 × 10^−2^	2.7 × 10^−2^
16–21 years	65.9	1.73	519	1	143	284	1.5 × 10^−2^	2.5 × 10^−2^
21–30 years	71.9	1.81	543	1	138	272	1.4 × 10^−2^	2.4 × 10^−2^
30–40 years	74.8	1.85	555	1	135	267	1.4 × 10^−2^	2.4 × 10^−2^

^a^ Body weight (BW) and ^b^ Total body area (SA) from the USA’s *EPA’s Exposure Factors Handbook* (EPA 2011 [54], https://assessments.epa.gov/risk/document/&deid=236252#downloads, accessed on 20 March 2026).

**Table 3:** Summaries of studies on dermal exposure to OPFRs.

Products/ Environment	Groups	Number of OPFRs	Concentration	Daily Exposure (ng·kg^−1^·day^−1^)	Reference
Cosmetic sponges	Females (11–40 years)	25	15.5–64.7 ng·cm^−2^ (wet)	267–304	This work
Electronic waste	Workers	13	Median: 183 ng (per handwipe)	2.07 (TPhP)	[25]
Toys	Children	18	Range: 6.82–228,254 ng·g^−1^	21.4–52.4	[39]
Polyurethane foam mats	Children	16	Range: 4100–29,000 ng·g^−1^	4890	[61]
Play mats	Children	9	Range: 6.6–7400 ng·g^−1^	0.024–0.029	[49]
Textiles	Children	20	4.85–1,180,000 ng·g^−1^	0.92–1.13	[50]
Kindergartens	Children	9	Median: 1840 ng·m^−2^ (forehead wipe) 205 ng·m^−2^ (toys wipe) 84.4 ng·m^−2^ (desk wipe)	3710	[51]
Haze	College students	10	Mean: 7400 ± 1600 ng·m^−2^ (forehead wipe)	6.7	[52]
Indoor air of automobile parts shops	Workers > 16 years old	15	Mean: 258 ng·m^−3^	279	[53]

**Table 4:** Recoveries and RSD at different spiked levels of 25 OPFRs extracted from Strat-M^TM^ (n = 3).

Analytes	LOD (mg·kg^−1^)	LOQ (mg·kg^−1^)	Spiked Levels					
			0.5 mg·kg^−1^		2.5 mg·kg^−1^		5 mg·kg^−1^	
			Recovery (%)	RSD (%)	Recovery (%)	RSD (%)	Recovery (%)	RSD (%)
TMP	0.05	0.10	106	5.92	99.6	4.73	79.1	2.66
TEP	0.02	0.05	104	2.96	118	3.03	86.6	1.79
TiPP	0.02	0.05	109	1.50	107	3.34	81.0	1.12
TPrP	0.02	0.05	111	1.88	120	1.81	86.1	1.79
TiBP	0.02	0.05	106	0.86	116	0.31	84.7	1.23
TnBP	0.02	0.05	74.7	7.48	118	4.82	85.9	3.10
TPeP	0.02	0.05	102	1.49	106	1.86	86.1	0.92
THP	0.02	0.05	103	2.95	99.3	0.30	91.8	1.36
TBEP	0.02	0.05	113	2.71	106	1.97	81.3	1.61
TEHP	0.02	0.05	82.5	6.10	103	3.95	79.5	1.37
TPhP	0.10	0.30	125	3.72	101	2.52	75.	2.65
TXP	0.02	0.05	103	1.67	106	8.22	78.0	7.16
TDMPP	0.02	0.05	104	3.60	103	6.94	77.9	4.48
EHDPP	0.02	0.05	80.5	12.1	103	7.09	73.6	5.76
MDPP	0.10	0.30	125	4.46	102	3.17	78.9	1.22
TPPO	0.02	0.05	116	4.98	112	3.91	87.1	0.59
TMCP	0.02	0.05	111	5.54	101	2.13	78.1	2.05
TOTP	0.02	0.05	129	3.27	102	2.59	69.8	3.19
RDP	0.02	0.05	107	2.75	98.9	1.81	79.1	0.95
BDP	0.02	0.05	117	7.45	93.7	11.5	75.1	15.4
TiPPP	0.05	0.10	115	4.26	99.6	3.44	80.7	4.23
TCEP	0.05	0.10	114	7.93	89.8	3.31	63.8	1.02
TCPP	0.20	0.50	110	7.82	111	5.66	82.9	2.08
TDCP	0.10	0.30	109	3.60	106	7.33	79.1	8.98
TDBPP	0.10	0.30	102	5.23	87.8	9.58	72.8	6.89

## Data Availability

No new code was generated in this study. The experimental data presented are available from the corresponding author upon reasonable request.

## Supplementary Materials

The following supporting information can be downloaded at: https://www.mdpi.com/article/10.3390/molecules31071067/s1, Table S1. Information of samples (n = 86) analyzed in this study. Table S2. Detection frequencies (%) and concentrations (ng·g^−1^) of OPFRs in samples with different color. Table S3. The detection frequency (%) and concentration (ng·g^−1^) of OPFRs in decolorized and non-decolorized samples during sample extraction. Table S4. OPFRs concentration (ng·g^−1^, mean ± SD) on the surface and inner core of powder puffs (n = 5) and beauty blenders (n = 5). Table S5. The water absorption capacity of cosmetic sponges in different states. Table S6. Area concentrations (ng·g^−1^, mean ± SD) of OPFRs assessed by simulated makeup process. Table S7. Daily exposure (ng·kg^−1^·day^−1^) to OPFRs for females aged from 11–40 years via dry cosmetic sponges. Table S8. Daily exposure (ng·kg^−1^·day^−1^) to OPFRs for females aged from 11–40 years via wet cosmetic sponges. Table S9. Information on the target OPFRs analyzed in this study. Table S10. Risk assessment to OPFRs for females aged from 11–40 years via dry cosmetic sponges. Table S11. Risk assessment to OPFRs for females aged from 11–40 years via wet cosmetic sponges. Table S12. MTT assay results using L-929 cell line for OPFRs solution prepared according to the exposure concentration. Table S13. MS parameters of 25 OPFRs. Table S14. Liquid chromatography gradient elution conditions. Figure S1. Heat map of OPFRs concentration in cosmetic sponges (normalized to maximum). Figure S2. Mean concentrations of OPFRs on the surface and in the core of the sample (a) powder puffs (n = 5); (b) beauty blenders (n = 5). Figure S3. The water absorption capacity of cosmetic sponges for states of (a) water absorption saturation, and (b) water squeeze. Figure S4. The migration of OPFRs for the wet cosmetic sponge (T-33) to be reused ten times.
