# Supplementary material for: Health Risks of Organophosphate Flame Retardants (OPFRs) in Facial Cosmetic Sponges via Dermal Exposure
Source: Molecules. 2026 Mar 24;31(7):1067. doi: 10.3390/molecules31071067 (PMC13074967; doi:10.3390/molecules31071067)
Supplement: Supplementary file 1 [file molecules-31-01067-s001.zip › molecules-4176133-supplementary.pdf]

Supporting information

Health Risks of Organophosphate Flame Retardants (OPFRs) in Facial Cosmetic Sponges via Dermal Exposure

Yang Yang [1,2,*], Yan Luo [1,2], Guiqin Liu [1], Jingfei Li [1,2], Xiangyong Meng [1], Cuicui Zheng [1], Zheng Zhang [1], Chun Yang [1,2], Jia Qiu [1,2] and Hui Cao [1,2]

[1] Zhejiang Institute of Quality Sciences, Hangzhou 310018, China
[2] Zhejiang Key Laboratory of Consumer Product Safety Research Under Provincial Market Supervision, Hangzhou 310018, China
* Correspondence: sober_yy@126.com

Table S1 Information of samples (n=86) analyzed in this study.

Sample number	Test number	Sample name	Color	Decolorization or not	Material composition
S-1	T-1	Beauty blender	Brown	Yes	/
S-2	T-2	Beauty powder puff	Light beige	No	Polyurethane
	T-3	Beauty powder puff	Yellow	No	Polyurethane
	T-4	Beauty powder puff	Green	No	Polyurethane
	T-5	Beauty powder puff	Brown	No	Polyurethane
	T-6	Beauty powder puff	Blue	No	Polyurethane
	T-7	Beauty powder puff	Pink	Yes	Polyurethane
	T-8	Beauty powder puff	Pink	Yes	Polyurethane
	T-9	Beauty powder puff	Red	No	Polyurethane
S-3	T-10	Beauty powder puff	Light beige	No	Styrene butadiene rubber
S-4	T-11	Beauty powder puff	Light beige	No	Polyurethane
S-5	T-12	Beauty blender	Yellow	No	Resin, seaweed
	T-13	Beauty blender	Yellow	No	Resin, seaweed
	T-14	Beauty blender	Green	No	Resin, seaweed
	T-15	Beauty blender	Red	Yes	Resin, seaweed
S-6	T-16	Beauty powder puff	Yellow	No	Styrene butadiene rubber
S-7	T-17	Beauty powder puff	Light beige	No	Polyurethane
	T-18	Beauty powder puff	Blue	No	Polyurethane
S-8	T-19	Beauty powder puff	Light beige	No	/
S-9	T-20	Beauty blender	Brown	No	/
	T-21	Beauty blender	Red	Yes	/
	T-22	Beauty blender	Green	No	/
S-10	T-23	Beauty blender	Green	No	Polyurethane
	T-24	Beauty blender	Green	No	Polyurethane
	T-25	Beauty blender	Green	No	Polyurethane
	T-26	Beauty blender	Blue	No	Polyurethane
S-11	T-27	Beauty blender	Purple	No	Polyurethane
	T-28	Beauty blender	Purple	Yes	Polyurethane
	T-29	Beauty blender	Purple	No	Polyurethane
	T-30	Beauty blender	Pink	No	Polyurethane
S-12	T-31	Thumb puff	Light beige	No	/
S-13	T-32	Beauty powder puff	Light beige	No	Polyurethane
S-14	T-33	Beauty powder puff	Light beige	No	Polyurethane
S-15	T-34	Beauty powder puff	Brown	No	/
S-16	T-35	Beauty powder puff	Light beige	No	Polyurethane
S-17	T-36	Beauty powder puff	Black	No	/
S-18	T-37	Beauty powder puff	Light beige	No	/
S-19	T-38	Beauty blender	Yellow	No	/

	T-39	Beauty blender	Green	No	/
S-20	T-40	Beauty blender	Pink	Yes	/
S-21	T-41	Beauty powder puff	Light beige	No	Latex
S-22	T-42	Beauty blender	Purple	No	Polyurethane
	T-43	Beauty blender	Pink	Yes	Polyurethane
S-23	T-44	Beauty powder puff	Purple	No	Polyurethane
	T-45	Beauty powder puff	Green	No	Polyurethane
	T-46	Beauty powder puff	Yellow	No	Polyurethane
S-24	T-47	Beauty powder puff	White	No	Polyurethane
	T-48	Beauty powder puff	Blue	No	Polyurethane
	T-49	Beauty powder puff	Brown	No	Polyurethane
S-25	T-50	Beauty powder puff	Purple	No	/
	T-51	Beauty powder puff	Red	Yes	/
	T-52	Beauty powder puff	Light beige	No	/
S-26	T-53	Beauty blender	White	No	/
S-27	T-54	Beauty powder puff	Light beige	No	/
S-28	T-55	Beauty powder puff	Yellow	No	/
S-29	T-56	Beauty blender	Light beige	No	Polyurethane
S-30	T-57	Beauty powder puff	Black	No	Polyurethane
S-31	T-58	Beauty powder puff	Pink	No	/
S-32	T-59	Beauty powder puff	Light beige	No	/
S-33	T-60	Beauty blender	Pink	No	Polyurethane
S-34	T-61	Triangular powder puff	Yellow	No	/
S-35	T-62	Triangular powder puff	Purple	No	/
S-36	T-63	Beauty powder puff	Blue	No	Polyurethane
S-37	T-64	Beauty powder puff	White	No	Polyurethane
S-38	T-65	Beauty powder puff	Light beige	No	Polyurethane
S-39	T-66	Beauty powder puff	White	No	Polyurethane
S-40	T-67	Beauty powder puff	Black	Yes	Latex
S-41	T-68	Beauty powder puff	Pink	No	Styrene butadiene rubber
S-42	T-69	Thumb puff	Yellow	No	/
S-43	T-70	Thumb puff	Black	No	Polyurethane
S-44	T-71	Beauty powder puff	Pink	Yes	/
	T-72	Beauty powder puff	Light beige	No	/
S-45	T-73	Beauty powder puff	Black	No	/
S-46	T-74	Beauty blender	Black	No	Polyurethane
S-47	T-75	Beauty blender	Yellow	No	Polyurethane
S-48	T-76	Beauty blender	Pink	No	Polyurethane
S-49	T-77	Beauty blender	Yellow	No	Polyurethane
S-50	T-78	Beauty blender	Blue	No	Polyurethane
S-51	T-79	Beauty blender	Light beige	No	Polyurethane
	T-80	Beauty blender	Purple	Yes	Polyurethane
S-52	T-81	Beauty blender	Brown	No	/
S-53	T-82	Beauty blender	White	No	Polyurethane
S-54	T-83	Thumb puff	Red	Yes	/

	T-84	Thumb puff	Green	Yes	/
	T-85	Thumb puff	Purple	No	/
	T-86	Thumb puff	Blue	No	/

Table S2 Detection frequencies (%) and concentrations (ng·g^{-1}) of OPFRs in samples with different color.

Analytes	Dark-colored (n=46)		Light-colored (n=40)		Brown(n=6)		Light beige (n=18)		Yellow (n=11)		Green (n=9)	
	DF (%)	Range (ng·g^{-1})	DF (%)	Range (ng·g^{-1})	DF (%)	Range (ng·g^{-1})	DF (%)	Range (ng·g^{-1})	DF (%)	Range (ng·g^{-1})	DF (%)	Range (ng·g^{-1})
TMP	0.00	ND	0.00	ND	0.00	ND	0.00	ND	0.00	ND	0.00	ND
TEP	19.6	ND-<LOQ	30.0	ND-285	33.3	ND-<LOQ	27.8	ND-<LOQ	27.27	ND-<LOQ	22.2	ND-285
TiPP	0.00	ND	3.85	ND-<LOQ	0.00	ND	5.56	ND-<LOQ	0.00	ND	0.00	ND
TPrP	0.00	ND	0.00	ND	0.00	ND	0.00	ND	0.00	ND	0.00	ND
TiBP	28.3	ND-9399	37.5	ND-3028	33.3	ND-9399	44.4	ND-2072	27.27	ND-3028	11.1	ND-107
TnBP	2.17	ND-<LOQ	5.00	ND-871	0.00	ND	5.56	ND-708	9.09	ND-870.8	0.00	ND
TPeP	0.00	ND	0.00	ND	0.00	ND	0.00	ND	0.00	ND	0.00	ND
THP	0.00	ND	0.00	ND	0.00	ND	0.00	ND	0.00	ND	0.00	ND
TBEP	8.70	ND-<LOQ	10.0	ND-101	0.00	ND	16.7	ND-101	9.09	ND-<LOQ	0.00	ND
TEHP	13.0	ND-<LOQ	12.5	ND-54.2	0.00	ND	11.1	ND-<LOQ	9.09	ND	0.00	ND
TPhP	50.0	ND-1556	40.0	ND-7196	16.7	ND-<LOQ	44.4	ND-7196	27.27	ND-740.3	44.4	ND-1016
TXP	0.00	ND	0.00	ND	0.00	ND	0.00	ND	0.00	ND	0.00	ND
TDMPP	0.00	ND	0.00	ND	0.00	ND	0.00	ND	0.00	ND	0.00	ND
EHDPP	17.4	ND-2057	15.0	ND-1041	16.7	ND-<LOQ	22.2	ND-1041	9.09	ND-723.3	0.00	ND
MDPP	0.00	ND	0.00	ND	0.00	ND	0.00	ND	0.00	ND	0.00	ND
TPPO	19.6	ND-369	7.50	ND-3128	33.3	ND-148	16.7	ND-3128	0.00	ND	22.2	ND-212
TMCP	0.00	ND	0.00	ND	0.00	ND	0.00	ND	0.00	ND	0.00	ND
TOTP	0.00	ND	0.00	ND	0.00	ND	0.00	ND	0.00	ND	0.00	ND
RDP	4.35	ND-153	0.00	N	0.00	ND	0.00	ND	0.00	ND	11.1	ND-<LOQ
BDP	0.00	ND	0.00	ND	0.00	ND	0.00	ND	0.00	ND	0.00	ND
TiPPP	0.00	ND	0.00	ND	0.00	ND	0.00	ND	0.00	ND	0.00	ND
TCEP	6.52	ND-<LOQ	0.00	ND	16.7	ND-<LOQ	0.00	ND	0.00	ND	0.00	ND
TCPP	6.52	ND-1252	12.5	ND-2386	16.7	ND-<LOQ	22.2	ND-1097	9.09	ND-2386	0.00	ND
TDCP	0.00	ND	0.00	ND	0.00	ND	0.00	ND	0.00	ND	0.00	ND
TDBPP	0.00	ND	0.00	ND	0.00	ND	0.00	ND	0.00	ND	0.00	ND
Σ alkyl-OPFRs	43.5	ND-9424	65.0	ND-3053	33.3	ND-9424	72.2	ND-2072	45.45	ND-3053	22.2	ND-285

Σ aryl-OPFRs	67.4	ND-2057	50.0	ND-7196	16.7	ND-148	66.7	ND-7196	27.27	ND-873.3	66.7	ND-1016
Σ Cl-Br-OPFRs	8.70	ND-1255	12.5	ND-2386	16.7	ND-275	22.2	ND-1097	9.09	ND-2386	0.00	ND
Σ OPFRs	76.1	ND-9624	75.0	ND-7496	50.0	ND-9424	77.8	ND-7496	63.64	ND-3310	77.8	ND-1016

Analytes	Blue (n=7)		Pink (n=10)		Red (n=5)		Purple (n=9)		Black (n=6)		White (n=5)	
	DF (%)	Range (ng·g^{-1})	DF (%)	Range (ng·g^{-1})	DF (%)	Range (ng·g^{-1})	DF (%)	Range (ng·g^{-1})	DF (%)	Range (ng·g^{-1})	DF (%)	Range (ng·g^{-1})
TMP	0.00	ND	0.00	ND	0.00	ND	0.00	ND	0.00	ND	0.00	ND
TEP	28.6	ND-<LOQ	10.0	ND-<LOQ	40.0	ND-<LOQ	22.2	ND-<LOQ	0.00	ND	60.0	ND-68.8
TiPP	0.00	ND	0.00	ND	0.00	ND	0.00	ND	0.00	ND	0.00	ND
TPrP	0.00	ND	0.00	ND	0.00	ND	0.00	ND	0.00	ND	0.00	ND
TiBP	28.6	ND-3715	20.0	ND-1811	40.0	ND-3585	33.3	ND-2031	33.3	ND-3043	60.0	ND-99.6
TnBP	0.00	ND	10.0	ND-<LOQ	0.00	ND	0.00	ND	0.00	ND	0.00	ND
TPeP	0.00	ND	0.00	ND	0.00	ND	0.00	ND	0.00	ND	0.00	ND
THP	0.00	ND	0.00	ND	0.00	ND	0.00	ND	0.00	ND	0.00	ND
TBEP	0.00	ND	20.0	ND-<LOQ	20.0	ND-<LOQ	0.00	ND	16.7	ND-<LOQ	0.00	ND
TEHP	14.3	ND-54.2	30.0	ND-<LOQ	0.00	ND	22.2	ND-<LOQ	16.7	ND-<LOQ	0.00	ND
TPhP	57.1	ND-1556	60.0	ND-1491	40.0	ND-<LOQ	88.9	ND-1349	16.7	ND-669	40.0	ND-3637
TXP	0.00	ND	0.00	ND	0.00	ND	0.00	ND	0.00	ND	0.00	ND
TDMPP	0.00	ND	0.00	ND	0.00	ND	0.00	ND	0.00	ND	0.00	ND
EHDPP	14.3	ND-<LOQ	30.0	ND-1379	0.00	ND	11.1	ND-<LOQ	33.3	ND-2057	40.0	ND-<LOQ
MDPP	0.00	ND	0.00	ND	0.00	ND	0.00	ND	0.00	ND	0.00	ND
TPPO	0.00	ND	20.0	ND-181	20.0	ND-306	11.1	ND-369	16.7	ND-123	0.00	ND
TMCP	0.00	ND	0.00	ND	0.00	ND	0.00	ND	0.00	ND	0.00	ND
TOTP	0.00	ND	0.00	ND	0.00	ND	0.00	ND	0.00	ND	0.00	ND
RDP	0.00	ND	10.0	ND-153	0.00	ND	0.00	ND	0.00	ND	0.00	ND
BDP	0.00	ND	0.00	ND	0.00	ND	0.00	ND	0.00	ND	0.00	ND
TiPPP	0.00	ND	0.00	ND	0.00	ND	0.00	ND	0.00	ND	0.00	ND
TCEP	0.00	ND	0.00	ND	20.0	ND-<LOQ	11.1	ND<LOQ	0.00	ND	0.00	ND
TCPP	0.00	ND	10.0	ND-1252	20.0	ND<LOQ	11.1	ND-<LOQ	0.00	ND	0.00	ND
TDCP	0.00	ND	0.00	ND	0.00	ND	0.00	ND	0.00	ND	0.00	ND

TDBPP	0.00	ND	0.00	ND	0.00	ND	0.00	ND	0.00	ND	0.00	ND
Σ alkyl-OPFRs	57.1	ND-3715	50.0	ND-1811	40.0	ND-3610	55.6	ND-2056	50.0	ND-3043	80.0	ND-162
Σ aryl-OPFRs	57.1	ND-1556	70.0	ND-1491	40.0	ND-306	88.9	ND-1742	66.7	ND-2057	40.0	ND-3662
Σ Cl-Br-OPFRs	0.00	ND	10.0	ND-1252	20.0	ND-275	11.1	ND-275	0.00	ND	0.00	ND
Σ OPFRs	71.4	ND-5271	70.0	ND-1730	60.0	ND-3610	100	ND-3799	100	ND-3043	80.0	ND-3824

Table S3 The detection frequency (%) and concentration (ng·g^{-1}) of OPFRs in decolorized and non-decolorized samples during sample extraction.

Analytes	Non-discolored (n=72)		Discolored (n=14)	
	DF (%)	Range (ng·g^{-1})	DF (%)	Range (ng·g^{-1})
TMP	0.00	ND	0.00	ND
TEP	25.0	ND-285	21.4	ND-<LOQ
TiPP	1.39	ND-50.0	0.00	ND
TPrP	0.00	ND	0.00	ND
TiBP	33.3	ND-9399	28.6	ND-3585
TnBP	4.17	ND-871	0.00	ND
TPeP	0.00	ND	0.00	ND
THP	0.00	ND	0.00	ND
TBEP	9.72	ND-100	7.14	ND-<LOQ
TEHP	12.5	ND-54.2	14.3	ND-<LOQ
TPhP	43.1	ND-7196	57.1	ND-1491
TXP	0.00	ND	0.00	ND
TDMPP	0.00	ND	0.00	ND
EHDPP	16.7	ND-2057	14.3	ND-<LOQ
MDPP	0.00	ND	0.00	ND
TPPO	13.9	ND-3128	14.3	ND-306
TMCP	0.00	ND	0.00	ND
TOTP	0.00	ND	0.00	ND
RDP	2.78	ND-153	0.00	ND
BDP	0.00	ND	0.00	ND
TiPPP	0.00	ND	0.00	ND
TCEP	2.78	ND-<LOQ	7.14	ND-<LOQ
TCPP	11.1	ND-2386	7.14	ND-<LOQ
TDCP	0.00	ND	0.00	ND
TDBPP	0.00	ND	0.00	ND
Σ alkyl-OPFRs	55.6	ND-9424	42.9	ND-3635
Σ aryl-OPFRs	56.9	ND-7196	71.4	ND-1491
Σ Cl-Br-OPFRs	11.1	ND-2386	7.14	ND-275
Σ OPFRs	76.4	ND-9624	71.4	ND-3785

Table S4 OPFRs concentration (ng·g^{-1}, mean ± SD) on the surface and inner core of powder puffs (n=5) and beauty blenders (n=5).

Types		TEP	TiBP	TEHP	TPhP	EHDPP	TPPO	TCEP	TCPP
Powder puffs									
T-17	Surf.	ND	628±11.3	ND	1380±42.0	ND	ND	ND	ND
	Inn.	ND	781±129	ND	948±26.3	ND	ND	ND	ND
T-31	Surf.	31.6±6.25	31.0±2.14	ND	409±19.0	ND	3078±206	ND	ND
	Inn.	124±1.20	94.7±0.56	ND	312±53.8	ND	5017±97.1	ND	ND
T-32	Surf.	39.5±3.04	43.2±7.73	ND	6821±609	23.4±0.96	29.7±1.02	ND	ND
	Inn.	37.0±0.62	47.2±2.48	ND	6294±97.3	ND	ND	ND	ND
T-50	Surf.	10.1±0.19	9851±570	19.0±0.36	6.69±0.81	ND	ND	ND	ND
	Inn.	9.39±1.87	9583±125	ND	ND	ND	ND	ND	ND
T-52	Surf.	51.8±5.74	3944±625	ND	6.90±1.00	14.5±0.47	ND	ND	ND
	Inn.	22.4±0.31	3027±238	ND	ND	ND	ND	ND	ND
Mean	Surf.	26.6	2899	3.80	1725	7.58	621	ND	ND
	Inn.	38.5	2707	ND	1511	ND	1003	ND	ND
Beauty blenders									
T-23	Surf.	ND	ND	ND	2530±260	ND	ND	ND	ND
	Inn.	ND	ND	ND	396±22.7	ND	ND	ND	ND
T-44	Surf.	ND	56.1±12.6	43.6±0.99	725±105	ND	ND	ND	ND
	Inn.	ND	83.2±17.3	48.0±3.58	537±120	ND	ND	ND	ND
T-45	Surf.	12.4±0.80	4.69±0.63	48.4±5.18	705±41.8	ND	ND	10.4±0.72	ND
	Inn.	8.28±1.75	32.0±1.95	51.8±0.31	360±10.5	ND	ND	ND	ND
T-56	Surf.	ND	12.5±4.40	ND	28.6±0.74	829±103	ND	ND	2133±75.7
	Inn.	ND	9.39±0.96	ND	ND	8.11±2.77	ND	ND	103±18.8
T-60	Surf.	1.60±0.22	43.7±7.24	ND	ND	1292±334	1069±209	ND	976±147
	Inn.	9.20±1.08	37.0±0.87	ND	ND	158±41.8	1373±371	ND	388±107
Mean	Surf.	2.80	23.4	18.4	798	424	214	2.09	622
	Inn.	3.50	32.3	20.0	251	33.2	275	ND	98.2

Surf. refers to the surface
Inn. refers to the inner core

Table S5 The water absorption capacity of cosmetic sponges in different states.

Samples	Saturated water content ($g \cdot g^{-1}$)			Water content after squeezing ($g \cdot g^{-1}$)		
	Range	Mean	Median	Range	Mean	Median
Beauty blenders (n=20)	6.93-10.40	8.68	8.74	1.29-1.97	1.67	1.67
Power puffs (n=20)	3.53-6.63	5.27	4.92	0.77-1.59	1.09	1.08

Table S6 Area concentrations (ng·g^{-1}, mean ± SD) of OPFRs assessed by simulated makeup process.

Samples	Analytes	TEP	TiBP	TBEP	TEHP	TPhP	EHDPP	TPPO	TCPP	Σalkyl-OPFRs	Σ aryl-OPFRs	Σ Cl-Br-OPFRs	Σ OPFRs
T-17	C (ng·g^{-1})	ND	669±72.9	ND	ND	1490±203	ND	ND	ND	669	1490	ND	2159
	MR-D(ng·cm^{-2})	ND	1.10±4.16	ND	ND	9.62±0.77	ND	ND	ND	1.10	9.62	ND	10.7
	MR-W(ng·cm^{-2})	ND	14.4±1.31	ND	ND	14.5±2.06	ND	ND	ND	14.4	14.5	ND	28.8
T-31	C (ng·g^{-1})	<LOQ	<LOQ	ND	ND	337±57.0	ND	3128±447	ND	<LOQ	3465	ND	3515
	MR-D(ng·cm^{-2})	1.34±0.99	ND	ND	ND	14.5±2.14	ND	<LOQ	ND	1.34	14.7	ND	16.0
	MR-W(ng·cm^{-2})	0.84±0.30	1.72±1.11	ND	ND	7.06±0.26	ND	14.2±2.98	ND	2.56	21.2	ND	23.8
T-32	C (ng·g^{-1})	<LOQ	<LOQ	ND	ND	6226±603	ND	ND	ND	<LOQ	6226	ND	6276
	MR-D(ng·cm^{-2})	0.85±0.24	<LOQ	ND	ND	9.00±1.09	ND	ND	ND	1.41	9.00	ND	10.4
	MR-W(ng·cm^{-2})	1.07±0.11	1.17±0.38	ND	ND	26.7±2.53	ND	ND	ND	2.30	26.7	ND	29.0
T-33	C (ng·g^{-1})	<LOQ	<LOQ	ND	ND	7196±395	ND	ND	<LOQ	50.0	7196	250	7496
	MR-D(ng·cm^{-2})	1.20±0.26	ND	ND	ND	16.0±0.97	ND	ND	ND	1.20	16.0	ND	17.2
	MR-W(ng·cm^{-2})	0.85±0.18	4.36±0.41	ND	ND	41.7±3.52	ND	ND	<LOQ	5.21	41.7	5.57	52.5
T-50	C (ng·g^{-1})	<LOQ	9399±490	ND	ND	<LOQ	<LOQ	<LOQ	ND	9424	200	ND	9624
	MR-D(ng·cm^{-2})	<LOQ	21.1±1.09	ND	ND	8.86±0.71	ND	ND	ND	21.7	8.86	ND	30.6
	MR-W(ng·cm^{-2})	1.00±0.12	5.72±0.68	ND	ND	7.59±1.02	<LOQ	<LOQ	ND	6.72	8.70	ND	15.4
T-51	C (ng·g^{-1})	<LOQ	2031±234	ND	ND	1349±198	13.2±0.87	369±70.2	ND	2056	1731	ND	3787
	MR-D(ng·cm^{-2})	<LOQ	7.68±0.40	ND	ND	6.08±1.32	ND	ND	ND	8.24	6.08	ND	14.3
	MR-W(ng·cm^{-2})	<LOQ	34.8±4.57	ND	ND	9.56±3.09	ND	<LOQ	ND	35.4	10.1	ND	45.5
T-52	C (ng·g^{-1})	<LOQ	3585±166	<LOQ	ND	<LOQ	ND	ND	ND	3635	150	ND	3785
	MR-D(ng·cm^{-2})	1.03±0.20	19.0±1.74	ND	ND	13.9±2.20	ND	ND	ND	20.1	13.9	ND	34.0
	MR-W(ng·cm^{-2})	0.82±0.17	33. 9±3.85	<LOQ	ND	6.99±1.09	ND	ND	ND	35.3	6.99	ND	42.3
T-56	C (ng·g^{-1})	ND	<LOQ	<LOQ	ND	<LOQ	723±69.7	ND	2386±97.2	75.0	873	2386	3335
	MR-D(ng·cm^{-2})	ND	ND	ND	ND	13.8±2.12	ND	ND	ND	ND	13.8	ND	13.8

	MR-W(ng·cm^{-2})	ND	2.88±0.67	<LOQ	ND	12.8±3.79	<LOQ	ND	<LOQ	3.47	13.4	5.57	22.4
T-60	C (ng·g^{-1})	<LOQ	<LOQ	<LOQ	<LOQ	<LOQ	1041±252	1340±226	1097±75.3	75.0	2381	1097	3553
	MR-D(ng·cm^{-2})	<LOQ	ND	<LOQ	ND	7.51±1.04	<LOQ	2.19±0.77	<LOQ	1.12	10.3	5.57	17.0
	MR-W(ng·cm^{-2})	0.88±0.13	11.7±0.97	<LOQ	<LOQ	10.6±2.39	<LOQ	32.4±5.82	7.41±4.63	13.7	43.5	7.41	64.7

Note: C: concentration; MR-D: Migration measured with a dry sponge; MR-W: Migration measured with a wet sponge; ND: not detected.

Table S7 Daily exposure (ng·kg^{-1}·day^{-1}) to OPFRs for females aged from 11-40 years via dry cosmetic sponges.

Analytes	T-17				T-31				T-32			
	11-16 years	16-21 years	21-30 years	30-40 years	11-16 years	16-21 years	21-30 years	30-40 years	11-16 years	16-21 years	21-30 years	30-40 years
TEP	0.00	0.00	0.00	0.00	11.3	10.5	10.1	9.93	7.15	6.69	6.41	6.30
TiBP	9.27	8.66	8.31	8.16	0.00	0.00	0.00	0.00	4.70	4.39	4.21	4.14
TBEP	0.00	0.00	0.00	0.00	0.00	0.00	0.00	0.00	0.00	0.00	0.00	0.00
TEHP	0.00	0.00	0.00	0.00	0.00	0.00	0.00	0.00	0.00	0.00	0.00	0.00
TPhP	81.1	75.8	72.7	71.4	122	114	110	108	75.8	70.9	68.0	66.8
EHDPP	0.00	0.00	0.00	0.00	0.00	0.00	0.00	0.00	0.00	0.00	0.00	0.00
TPPO	0.00	0.00	0.00	0.00	4.72	4.41	4.23	4.16	0.00	0.00	0.00	0.00
TCPP	0.00	0.00	0.00	0.00	0.00	0.00	0.00	0.00	0.00	0.00	0.00	0.00
Σ alkyl-OPFRs	9.27	8.66	8.31	8.16	11.3	10.5	10.1	9.93	11.9	11.1	10.6	10.4
Σ aryl-OPFRs	81.1	75.8	72.7	71.4	124	116	111	109	75.8	70.9	68.0	66.8
Σ Cl-Br-OPFRs	0.00	0.00	0.00	0.00	0.00	0.00	0.00	0.00	0.00	0.00	0.00	0.00
Σ OPFRs	90.4	84.5	81.0	79.6	135	127	121	119	87.7	82.0	78.6	77.2

Analytes	T-33				T-50				T-51			
	11-16 years	16-21 years	21-30 years	30-40 years	11-16 years	16-21 years	21-30 years	30-40 years	11-16 years	16-21 years	21-30 years	30-40 years
TEP	10.1	9.46	9.07	8.91	4.70	4.39	4.21	4.14	4.70	4.39	4.21	4.14
TiBP	0.00	0.00	0.00	0.00	178	167	160	157	64.7	60.5	58.0	57.0
TBEP	0.00	0.00	0.00	0.00	0.00	0.00	0.00	0.00	0.00	0.00	0.00	0.00
TEHP	0.00	0.00	0.00	0.00	0.00	0.00	0.00	0.00	0.00	0.00	0.00	0.00
TPhP	135	126	121	119	74.6	69.8	66.9	65.7	51.2	47.9	45.9	45.1
EHDPP	0.00	0.00	0.00	0.00	0.00	0.00	0.00	0.00	0.00	0.00	0.00	0.00
TPPO	0.00	0.00	0.00	0.00	0.00	0.00	0.00	0.00	0.00	0.00	0.00	0.00
TCPP	0.00	0.00	0.00	0.00	0.00	0.00	0.00	0.00	0.00	0.00	0.00	0.00

Σ alkyl-OPFRs	10.1	9.46	9.07	8.91	183	171	164	161	69.4	64.9	62.2	61.1
Σ aryl-OPFRs	135	126	121	119	74.6	69.8	66.9	65.7	51.2	47.9	45.9	45.1
Σ Cl-Br-OPFRs	0.00	0.00	0.00	0.00	0.00	0.00	0.00	0.00	0.00	0.00	0.00	0.00
Σ OPFRs	145	135	130	128	258	241	231	227	121	113	108	106

Analytes	T-52				T-56				T-60			
	11-16 years	16-21 years	21-30 years	30-40 years	11-16 years	16-21 years	21-30 years	30-40 years	11-16 years	16-21 years	21-30 years	30-40 years
TEP	8.67	8.11	7.77	7.64	0.00	0.00	0.00	0.00	4.70	4.39	4.21	4.14
TiBP	160	150	144	141	0.00	0.00	0.00	0.00	0.00	0.00	0.00	0.00
TBEP	0.00	0.00	0.00	0.00	0.00	0.00	0.00	0.00	4.72	4.41	4.23	4.16
TEHP	0.00	0.00	0.00	0.00	0.00	0.00	0.00	0.00	0.00	0.00	0.00	0.00
TPhP	117	110	105	103	116	108	104	102	63.3	59.2	56.7	55.7
EHDPP	0.00	0.00	0.00	0.00	0.00	0.00	0.00	0.00	4.70	4.39	4.21	4.14
TPPO	0.00	0.00	0.00	0.00	0.00	0.00	0.00	0.00	18.5	17.3	16.5	16.3
TCPP	0.00	0.00	0.00	0.00	0.00	0.00	0.00	0.00	47.0	43.9	42.1	41.4
Σ alkyl-OPFRs	169	158	152	149	0.00	0.00	0.00	0.00	9.41	8.80	8.44	8.29
Σ aryl-OPFRs	117	110	105	103	116	108	104	102	86.4	80.8	77.5	76.1
Σ Cl-Br-OPFRs	0.00	0.00	0.00	0.00	0.00	0.00	0.00	0.00	47.0	43.9	42.1	41.4
Σ OPFRs	286	268	257	252	116	108	104	102	143	134	128	126

Table S8 Daily exposure (ng·kg^{-1}·day^{-1}) to OPFRs for females aged from 11-40 years via wet cosmetic sponges.

Analytes	T-17				T-31				T-32			
	11-16 years	16-21 years	21-30 years	30-40 years	11-16 years	16-21 years	21-30 years	30-40 years	11-16 years	16-21 years	21-30 years	30-40 years
TEP	0.00	0.00	0.00	0.00	7.12	6.65	6.38	6.27	9.01	8.42	8.08	7.94
TiBP	121	113	108	107	14.5	13.5	13.0	12.7	9.86	9.22	8.84	8.69
TBEP	0.00	0.00	0.00	0.00	0.00	0.00	0.00	0.00	0.00	0.00	0.00	0.00
TEHP	0.00	0.00	0.00	0.00	0.00	0.00	0.00	0.00	0.00	0.00	0.00	0.00
TPhP	122	114	109	107	59.5	55.6	53.3	52.4	225	210	202	198
EHDPP	0.00	0.00	0.00	0.00	0.00	0.00	0.00	0.00	0.00	0.00	0.00	0.00
TPPO	0.00	0.00	0.00	0.00	120	112	107	105	0.00	0.00	0.00	0.00
TCPP	0.00	0.00	0.00	0.00	0.00	0.00	0.00	0.00	0.00	0.00	0.00	0.00
Σ alkyl-OPFRs	121	113	108	107	21.6	20.2	19.3	19.0	19.4	18.1	17.4	17.0
Σ aryl-OPFRs	122	114	109	107	179	167	160	158	225	210	202	198
Σ Cl-Br-OPFRs	0.00	0.00	0.00	0.00	0.00	0.00	0.00	0.00	0.00	0.00	0.00	0.00
Σ OPFRs	243	227	217	214	201	187	179	177	244	228	219	215
Analytes	T-33				T-50				T-51			
	11-16 years	16-21 years	21-30 years	30-40 years	11-16 years	16-21 years	21-30 years	30-40 years	11-16 years	16-21 years	21-30 years	30-40 years
TEP	7.16	6.69	6.42	6.31	8.41	7.86	7.54	7.41	4.70	4.39	4.21	4.14
TiBP	36.8	34.4	33.0	32.4	48.2	45.1	43.2	42.5	293	274	263.0	258
TBEP	0.00	0.00	0.00	0.00	0.00	0.00	0.00	0.00	0.00	0.00	0.00	0.00
TEHP	0.00	0.00	0.00	0.00	0.00	0.00	0.00	0.00	0.00	0.00	0.00	0.00
TPhP	351	328	315	309	63.9	59.8	57.3	56.3	80.6	75.3	72.2	70.9
EHDPP	0.00	0.00	0.00	0.00	4.70	4.39	4.21	4.14	0.00	0.00	0.00	0.00
TPPO	0.00	0.00	0.00	0.00	4.70	4.39	4.21	4.14	4.70	4.39	4.21	4.14

TCPP	47.0	43.9	42.1	41.4	0.00	0.00	0.00	0.00	0.00	0.00	0.00	0.00
Σ alkyl-OPFRs	43.9	41.1	39.4	38.7	56.6	52.9	50.8	49.9	298	279	267	263
Σ aryl-OPFRs	351	328	315	309	73.3	68.5	65.7	64.6	85.3	79.7	76.4	75.1
Σ Cl-Br-OPFRs	47.0	43.9	42.1	41.4	0.00	0.00	0.00	0.00	0.00	0.00	0.00	0.00
Σ OPFRs	442	413	397	389	130	121	117	115	383	359	343	338

Analytes	T-52				T-56				T-60			
	11-16 years	16-21 years	21-30 years	30-40 years	11-16 years	16-21 years	21-30 years	30-40 years	11-16 years	16-21 years	21-30 years	30-40 years
TEP	6.88	6.43	6.17	6.06	0.00	0.00	0.00	0.00	7.40	6.92	6.64	6.52
TiBP	286	267	256	251	24.3	22.7	21.8	21.4	99.0	92.4	88.6	87.1
TBEP	4.70	4.39	4.21	4.14	4.70	4.39	4.21	4.14	4.70	4.39	4.21	4.14
TEHP	0.00	0.00	0.00	0.00	0.00	0.00	0.00	0.00	4.70	4.39	4.21	4.14
TPhP	58.9	55.1	52.8	51.9	108	101	96.9	95.2	88.0	83.2	79.7	78.3
EHDPP	0.00	0.00	0.00	0.00	4.70	4.39	4.21	4.14	4.70	4.39	4.21	4.14
TPPO	0.00	0.00	0.00	0.00	0.00	0.00	0.00	0.00	273	255	245	241
TCPP	0.00	0.00	0.00	0.00	47.0	43.9	42.1	41.4	62.5	58.4	56.0	55.0
Σ alkyl-OPFRs	297	278	266	262	29.3	27.3	26.2	25.8	116	108	104	102
Σ aryl-OPFRs	58.9	55.1	52.8	51.9	113	105	101	99.3	367	343	329.0	323
Σ Cl-Br-OPFRs	0.00	0.00	0.00	0.00	47.0	43.9	42.1	41.4	62.5	58.4	56.0	55.0
Σ OPFRs	356	333	319	314	189	176	169	167	546	509	489	480

Table S9 Information on the target OPFRs analyzed in this study.

Abbreviation	CAS number	Molecular formula	MW (g·mol^{-1})	log Kow [a]
TMP	512-56-1	$C_3H_9O_4P$	140.1	-0·60
TEP	78-40-0	$C_6H_{15}O_4P$	182.2	0.87
TPrP	513-08-6	$C_9H_{21}O_4P$	224.2	2.35
TiPP	513-02-0	$C_9H_{21}O_4P$	224.2	2.12
TnBP	126-73-8	$C_{12}H_{27}O_4P$	266.3	3.82
TiBP	126-71-6	$C_{12}H_{27}O_4P$	266.3	3.60
THP	2528-39-4	$C_{18}H_{39}O_4P$	350.5	6.76
TEHP	78-42-2	$C_{24}H_{51}O_4P$	434.6	9.49
TPEP	2528-38-3	$C_{15}H_{33}O_4P$	308.4	5.29
TBEP	78-51-3	$C_{18}H_{39}O_7P$	398.5	3.00
TPhP	115-86-6	$C_{18}H_{15}O_4P$	326.3	4.70
TiPPP	68937-41-7	$C_{27}H_{33}O_4P$	452.5	9.07
TXP	25155-23-1	$C_{24}H_{27}O_4P$	410.4	7.98
TPPO	791-28-6	$C_{18}H_{15}OP$	278.3	2.87
EHDPP	1241-94-7	$C_{20}H_{27}O_4P$	362.4	6.30
MDPP	26444-49-5	$C_{19}H_{17}O_4P$	340.4	5.25
RDP	57583-54-7	$C_{30}H_{24}O_8P_2$	574.5	7.41
BDP	5945-33-5	$C_{39}H_{34}O_8P_2$	692.6	10.02
TMCP	563-04-2	$C_{21}H_{21}O_4P$	368.4	6.34
TOTP	78-30-8	$C_{21}H_{21}O_4P$	368.4	6.34
TDMPP	85417-41-0	$C_{24}H_{27}O_6P$	442.4	5.51
TCEP	115-96-8	$C_6H_{12}Cl_3O_4P$	285.5	1.63
TCPP	13674-84-5	$C_9H_{18}Cl_3O_4P$	327.6	2.89
TDCP	13674-87-8	$C_9H_{15}Cl_6O_4P$	430.9	3.65
TDBPP	126-72-7	$C_9H_{15}Br_6O_4P$	697.6	4.19

[a] log Kow calculated by EPI Suit^{TM} v4.1

Table S10 Risk assessment to OPFRs for females aged from 11-40 years via dry cosmetic sponges.

Analytes	T-17				T-31				T-32			
	11-16 years	16-21 years	21-30 years	30-40 years	11-16 years	16-21 years	21-30 years	30-40 years	11-16 years	16-21 years	21-30 years	30-40 years
TEP	-	-	-	-	9.0×10^{-5}	8.4×10^{-5}	8.1×10^{-5}	7.9×10^{-5}	5.7×10^{-5}	5.3×10^{-5}	5.1×10^{-5}	5.0×10^{-5}
TiBP	9.3×10^{-5}	8.7×10^{-5}	8.3×10^{-5}	8.2×10^{-5}	-	-	-	-	4.7×10^{-5}	4.4×10^{-5}	4.2×10^{-5}	4.1×10^{-5}
TBEP	-	-	-	-	-	-	-	-	-	-	-	-
TEHP	-	-	-	-	-	-	-	-	-	-	-	-
TPhP	1.2×10^{-2}	1.1×10^{-2}	1.0×10^{-2}	1.0×10^{-2}	1.7×10^{-2}	1.6×10^{-2}	1.6×10^{-2}	1.5×10^{-2}	1.1×10^{-2}	1.0×10^{-2}	9.7×10^{-3}	9.5×10^{-3}
EHDPP	-	-	-	-	-	-	-	-	-	-	-	-
TCPP	-	-	-	-	-	-	-	-	-	-	-	-
Σ alkyl-OPFRs	9.3×10^{-5}	8.7×10^{-5}	8.3×10^{-5}	8.2×10^{-5}	9.0×10^{-5}	8.4×10^{-5}	8.1×10^{-5}	7.9×10^{-5}	1.0×10^{-4}	9.7×10^{-5}	9.3×10^{-5}	9.2×10^{-5}
Σ aryl-OPFRs	1.2×10^{-2}	1.1×10^{-2}	1.0×10^{-2}	1.0×10^{-2}	1.8×10^{-2}	1.7×10^{-2}	1.6×10^{-2}	1.6×10^{-2}	1.1×10^{-2}	1.0×10^{-2}	9.7×10^{-3}	9.5×10^{-3}
Σ Cl-Br-OPFRs	-	-	-	-	-	-	-	-	-	-	-	-
Σ OPFRs	1.2×10^{-2}	1.1×10^{-2}	1.0×10^{-2}	1.0×10^{-2}	1.8×10^{-2}	1.7×10^{-2}	1.6×10^{-2}	1.6×10^{-2}	1.1×10^{-2}	1.0×10^{-2}	9.8×10^{-3}	9.6×10^{-3}

Analytes	T-33				T-50				T-51			
	11-16 years	16-21 years	21-30 years	30-40 years	11-16 years	16-21 years	21-30 years	30-40 years	11-16 years	16-21 years	21-30 years	30-40 years
TEP	8.1×10^{-5}	7.6×10^{-5}	7.3×10^{-5}	7.1×10^{-5}	3.8×10^{-5}	3.5×10^{-5}	3.4×10^{-5}	3.3×10^{-5}	3.8×10^{-5}	3.5×10^{-5}	3.4×10^{-5}	3.3×10^{-5}
TiBP	-	-	-	-	1.8×10^{-3}	1.7×10^{-3}	1.6×10^{-3}	1.6×10^{-3}	6.5×10^{-4}	6.0×10^{-4}	5.8×10^{-4}	5.7×10^{-4}
TBEP	-	-	-	-	-	-	-	-	-	-	-	-
TEHP	-	-	-	-	-	-	-	-	-	-	-	-
TPhP	1.9×10^{-2}	1.8×10^{-2}	1.7×10^{-2}	1.7×10^{-2}	1.1×10^{-2}	1.0×10^{-2}	9.6×10^{-3}	9.4×10^{-3}	7.3×10^{-3}	6.8×10^{-3}	6.6×10^{-3}	6.4×10^{-3}
EHDPP	-	-	-	-	-	-	-	-	-	-	-	-
TCPP	-	-	-	-	-	-	-	-	-	-	-	-
Σ alkyl-OPFRs	8.1×10^{-5}	7.6×10^{-5}	7.3×10^{-5}	7.1×10^{-5}	1.8×10^{-3}	1.7×10^{-3}	1.6×10^{-3}	1.6×10^{-3}	6.8×10^{-4}	6.4×10^{-4}	6.1×10^{-4}	6.0×10^{-4}

Σ aryl-OPFRs	1.9×10^{-2}	1.8×10^{-2}	1.7×10^{-2}	1.7×10^{-2}	1.1×10^{-2}	1.0×10^{-2}	9.6×10^{-3}	9.4×10^{-3}	7.3×10^{-3}	6.8×10^{-3}	6.6×10^{-3}	6.4×10^{-3}
Σ Cl-Br-OPFRs	-	-	-	-	-	-	-	-	-	-	-	-
Σ OPFRs	1.9×10^{-2}	1.8×10^{-2}	1.7×10^{-2}	1.7×10^{-2}	1.2×10^{-2}	1.2×10^{-2}	1.1×10^{-2}	1.1×10^{-2}	8.0×10^{-3}	7.5×10^{-3}	7.2×10^{-3}	7.0×10^{-3}

Analytes	T-52				T-56				T-60			
	11-16 years	16-21 years	21-30 years	30-40 years	11-16 years	16-21 years	21-30 years	30-40 years	11-16 years	16-21 years	21-30 years	30-40 years
TEP	6.9×10^{-5}	6.5×10^{-5}	6.2×10^{-5}	6.1×10^{-5}	-	-	-	-	3.8×10^{-5}	3.5×10^{-5}	3.4×10^{-5}	3.3×10^{-5}
TiBP	1.6×10^{-3}	1.5×10^{-3}	1.4×10^{-3}	1.4×10^{-3}	-	-	-	-	-	-	-	-
TBEP	-	-	-	-	-	-	-	-	2.4×10^{-4}	2.2×10^{-4}	2.1×10^{-4}	2.1×10^{-4}
TEHP	-	-	-	-	-	-	-	-	-	-	-	-
TPhP	1.7×10^{-2}	1.6×10^{-2}	1.5×10^{-2}	1.5×10^{-2}	1.7×10^{-2}	1.5×10^{-2}	1.5×10^{-2}	1.5×10^{-2}	9.0×10^{-3}	8.4×10^{-3}	8.1×10^{-3}	8.0×10^{-3}
EHDPP	-	-	-	-	-	-	-	-	7.8×10^{-3}	7.3×10^{-3}	7.0×10^{-3}	6.9×10^{-3}
TCPP	-	-	-	-	-	-	-	-	1.3×10^{-2}	1.2×10^{-2}	1.2×10^{-2}	1.1×10^{-2}
Σ alkyl-OPFRs	1.7×10^{-3}	1.6×10^{-3}	1.5×10^{-3}	1.5×10^{-3}	-	-	-	-	2.7×10^{-4}	2.6×10^{-4}	2.5×10^{-4}	2.4×10^{-4}
Σ aryl-OPFRs	1.7×10^{-2}	1.6×10^{-2}	1.5×10^{-2}	1.5×10^{-2}	1.7×10^{-2}	1.5×10^{-2}	1.5×10^{-2}	1.5×10^{-2}	1.8×10^{-2}	1.7×10^{-2}	1.6×10^{-2}	1.6×10^{-2}
Σ Cl-Br-OPFRs	-	-	-	-	-	-	-	-	1.2×10^{-2}	1.1×10^{-2}	1.1×10^{-2}	1.0×10^{-2}
Σ OPFRs	1.8×10^{-2}	1.7×10^{-2}	1.6×10^{-2}	1.6×10^{-2}	1.7×10^{-2}	1.5×10^{-2}	1.5×10^{-2}	1.5×10^{-2}	3.0×10^{-2}	2.8×10^{-2}	2.7×10^{-2}	2.6×10^{-2}

Table S11 Risk assessment to OPFRs for females aged from 11-40 years via wet cosmetic sponges.

Analytes	T-17				T-31				T-32			
	11-16 years	16-21 years	21-30 years	30-40 years	11-16 years	16-21 years	21-30 years	30-40 years	11-16 years	16-21 years	21-30 years	30-40 years
TEP	-	-	-	-	5.7×10^{-5}	5.3×10^{-5}	5.1×10^{-5}	5.0×10^{-5}	7.2×10^{-5}	6.7×10^{-5}	6.5×10^{-5}	6.3×10^{-5}
TiBP	1.2×10^{-3}	1.1×10^{-3}	1.1×10^{-3}	1.1×10^{-3}	1.4×10^{-4}	1.4×10^{-4}	1.3×10^{-4}	1.3×10^{-4}	9.9×10^{-5}	9.2×10^{-5}	8.8×10^{-5}	8.7×10^{-5}
TBEP	-	-	-	-	-	-	-	-	-	-	-	-
TEHP	-	-	-	-	-	-	-	-	-	-	-	-
TPhP	1.7×10^{-2}	1.6×10^{-2}	1.6×10^{-2}	1.5×10^{-2}	8.5×10^{-3}	7.9×10^{-3}	7.6×10^{-3}	7.5×10^{-3}	3.2×10^{-2}	3.0×10^{-2}	2.9×10^{-2}	2.8×10^{-2}
EHDPP	-	-	-	-	-	-	-	-	-	-	-	-
TCPP	-	-	-	-	-	-	-	-	-	-	-	-
Σ alkyl-OPFRs	1.2×10^{-3}	1.1×10^{-3}	1.1×10^{-3}	1.1×10^{-3}	2.0×10^{-4}	1.9×10^{-4}	1.8×10^{-4}	1.8×10^{-4}	1.7×10^{-4}	1.6×10^{-4}	1.5×10^{-4}	1.5×10^{-4}
Σ aryl-OPFRs	1.7×10^{-2}	1.6×10^{-2}	1.6×10^{-2}	1.5×10^{-2}	8.0×10^{-3}	8.4×10^{-3}	7.6×10^{-3}	7.7×10^{-3}	3.2×10^{-2}	3.0×10^{-2}	2.9×10^{-2}	2.8×10^{-2}
Σ Cl-Br-OPFRs	-	-	-	-	-	-	-	-	-	-	-	-
Σ OPFRs	1.9×10^{-2}	1.7×10^{-2}	1.7×10^{-2}	1.6×10^{-2}	9.0×10^{-3}	8.4×10^{-3}	7.6×10^{-3}	7.7×10^{-3}	3.2×10^{-2}	3.0×10^{-2}	2.9×10^{-2}	2.8×10^{-2}

Analytes	T-33				T-50				T-51			
	11-16 years	16-21 years	21-30 years	30-40 years	11-16 years	16-21 years	21-30 years	30-40 years	11-16 years	16-21 years	21-30 years	30-40 years
TEP	5.7×10^{-5}	5.4×10^{-5}	5.1×10^{-5}	5.0×10^{-5}	6.7×10^{-5}	6.3×10^{-5}	6.0×10^{-5}	5.9×10^{-5}	3.8×10^{-5}	3.5×10^{-5}	3.4×10^{-5}	3.3×10^{-5}
TiBP	3.7×10^{-4}	3.4×10^{-4}	3.3×10^{-4}	3.2×10^{-4}	4.8×10^{-4}	4.5×10^{-4}	4.3×10^{-4}	4.2×10^{-4}	2.9×10^{-3}	2.7×10^{-3}	2.6×10^{-3}	2.6×10^{-3}
TBEP	-	-	-	-	-	-	-	-	-	-	-	-
TEHP	-	-	-	-	-	-	-	-	-	-	-	-
TPhP	5.0×10^{-2}	4.7×10^{-2}	4.5×10^{-2}	4.4×10^{-2}	9.1×10^{-3}	8.5×10^{-3}	8.2×10^{-3}	8.0×10^{-3}	1.2×10^{-2}	1.1×10^{-2}	1.0×10^{-2}	1.0×10^{-2}
EHDPP	-	-	-	-	7.8×10^{-3}	7.3×10^{-3}	7.0×10^{-3}	6.9×10^{-3}	4.5×10^{-6}	4.2×10^{-6}	4.0×10^{-6}	3.9×10^{-6}
TCPP	1.3×10^{-2}	1.2×10^{-2}	1.2×10^{-2}	1.1×10^{-2}	-	-	-	-	-	-	-	-
Σ alkyl-OPFRs	4.3×10^{-4}	4.0×10^{-4}	3.8×10^{-4}	3.7×10^{-4}	5.5×10^{-4}	5.1×10^{-4}	4.9×10^{-4}	4.8×10^{-4}	3.0×10^{-3}	2.8×10^{-3}	2.7×10^{-3}	2.6×10^{-3}
Σ aryl-OPFRs	5.0×10^{-2}	4.7×10^{-2}	4.5×10^{-2}	4.4×10^{-2}	1.7×10^{-2}	1.6×10^{-2}	1.5×10^{-2}	1.5×10^{-2}	1.2×10^{-2}	1.1×10^{-2}	1.1×10^{-2}	1.0×10^{-2}

Σ Cl-Br-OPFRs	1.3×10^{-2}	1.2×10^{-2}	1.2×10^{-2}	1.1×10^{-2}	-	-	-	-	-	-	-	-
Σ OPFRs	6.4×10^{-2}	5.9×10^{-2}	5.7×10^{-2}	5.6×10^{-2}	1.8×10^{-2}	1.7×10^{-2}	1.6×10^{-2}	1.6×10^{-2}	1.5×10^{-2}	1.4×10^{-2}	1.3×10^{-2}	1.3×10^{-2}

Analytes	T-52				T-56				T-60			
	11-16 years	16-21 years	21-30 years	30-40 years	11-16 years	16-21 years	21-30 years	30-40 years	11-16 years	16-21 years	21-30 years	30-40 years
TEP	5.5×10^{-5}	5.1×10^{-5}	4.9×10^{-5}	4.8×10^{-5}	-	-	-	-	5.9×10^{-5}	5.5×10^{-5}	5.3×10^{-5}	5.2×10^{-5}
TiBP	2.9×10^{-3}	2.7×10^{-3}	2.6×10^{-3}	2.5×10^{-3}	2.4×10^{-4}	2.3×10^{-4}	2.2×10^{-4}	2.1×10^{-4}	9.9×10^{-4}	9.2×10^{-4}	8.9×10^{-4}	8.7×10^{-4}
TBEP	2.3×10^{-4}	2.2×10^{-4}	2.1×10^{-4}	2.1×10^{-4}	2.3×10^{-4}	2.2×10^{-4}	2.1×10^{-4}	2.1×10^{-4}	2.3×10^{-4}	2.2×10^{-4}	2.1×10^{-4}	2.1×10^{-4}
TEHP	-	-	-	-	-	-	-	-	1.3×10^{-4}	1.3×10^{-4}	1.2×10^{-4}	1.2×10^{-4}
TPhP	8.4×10^{-3}	7.9×10^{-3}	7.5×10^{-3}	7.4×10^{-3}	1.5×10^{-2}	1.4×10^{-2}	1.4×10^{-2}	1.4×10^{-2}	1.3×10^{-2}	1.2×10^{-2}	1.1×10^{-2}	1.1×10^{-2}
EHDPP	-	-	-	-	7.8×10^{-3}	7.3×10^{-3}	7.0×10^{-3}	6.9×10^{-3}	7.8×10^{-3}	7.3×10^{-3}	7.0×10^{-3}	6.9×10^{-3}
TCPP	-	-	-	-	1.3×10^{-2}	1.2×10^{-2}	1.2×10^{-2}	1.1×10^{-2}	1.7×10^{-2}	1.6×10^{-2}	1.6×10^{-2}	1.5×10^{-2}
Σ alkyl-OPFRs	3.1×10^{-3}	2.9×10^{-3}	2.8×10^{-3}	2.8×10^{-3}	4.8×10^{-4}	4.5×10^{-4}	4.3×10^{-4}	4.2×10^{-4}	1.4×10^{-3}	1.3×10^{-3}	1.3×10^{-3}	1.2×10^{-3}
Σ aryl-OPFRs	8.4×10^{-3}	7.9×10^{-3}	7.5×10^{-3}	7.4×10^{-3}	2.3×10^{-2}	2.2×10^{-2}	2.1×10^{-2}	2.0×10^{-2}	2.0×10^{-2}	1.9×10^{-2}	1.9×10^{-2}	1.8×10^{-2}
Σ Cl-Br-OPFRs	-	-	-	-	1.3×10^{-2}	1.2×10^{-2}	1.2×10^{-2}	1.1×10^{-2}	1.7×10^{-2}	1.6×10^{-2}	1.6×10^{-2}	1.5×10^{-2}
Σ OPFRs	1.2×10^{-2}	1.1×10^{-2}	1.0×10^{-2}	1.0×10^{-2}	3.7×10^{-2}	3.4×10^{-2}	3.3×10^{-2}	3.2×10^{-2}	3.9×10^{-2}	3.7×10^{-2}	3.5×10^{-2}	3.5×10^{-2}

Table S12 MTT assay results using L-929 cell line for OPFRs solution prepared according to the exposure concentration

Samples	Mean ± SD	Cell viability (%)
Blank control	0.709±0.015	100.0
Negative control	0.673±0.027	94.9
Positive control	0.025±0.006	3.5
100% sample solution	0.024±0.002	3.4
75% sample solution	0.230±0.027	32.4
50% sample solution	0.424±0.019	59.8
25% sample solution	0.546±0.030	77.0

Table S13 MS parameters of 25 OPFRs [1]

Analyst	RT[a] (min)	Precursor ion (*m*/*z*)	Product ion (*m*/*z*)	DP[b] (V)	CE[c] (eV)
TMP	1.66	140.9	109.0*, 79.1	110	23, 28
TEP	1.99	183.1	99.0*, 127.0, 155.1	80	23, 15, 11
TiPP	2.75	225.1	99.0*, 141.0, 182.9	60	20, 12, 8
TPrP	2.99	225.1	99.1*, 141.0, 182.9	70	22, 12, 10
TiBP	4.94	267.3	99.0*, 155.0, 211.3	90	19, 12, 9
TnBP	5.08	267.1	99.0*, 154.7, 211.2	75	22, 15, 11
TPeP	7.02	309.3	99.0*, 169.0, 239.2	50	22, 15, 12
THP	8.45	351.2	99.1*, 267.2	70	22, 14
TBEP	5.54	399.3	198.9*,298.9	150	19, 17
TEHP	11.50	435.3	99.1*, 323.3, 211.0	90	14, 10, 14
TPhP	4.19	327.1	152.1*, 215.2, 250.9	150	47, 34, 34
TXP	8.03	411.2	194.0*, 178.9, 105.1	60	39, 52, 40
TDMPP	8.01	411.2	194.1*, 271.2, 178.9	85	41, 49, 42
EHDPP	6.87	363.1	251.0*, 77.1	60	13, 50
MDPP	4.94	341.0	152.1*, 165.0, 229.0	100	44, 38, 32
TPPO	2.76	279.2	201.1*, 173.1, 77.0	80	37, 43, 53
TMCP	6.32	369.1	166.1*, 91.0, 243.0	200	36, 38, 36
TOTP	6.23	369.1	165.9*, 91.1, 181.2	190	36, 37, 33
RDP	5.89	575.1	481.0*, 419.2, 405.2	50	46, 46, 51
BDP	7.63	693.1	367.1*, 327.1,	25	44, 39
TIPPP	8.50	453.3	327.0*, 369.5, 411.1	200	41, 32, 23
TCEP	2.07	285.0	62.9 *, 99.2, 223.0	140	45, 30, 17
TCPP	2.88	327.0	99.0*, 174.9, 250.8	100	25, 16, 12
TDCP	4.04	430.7	99.0*, 208.9, 320.9	170	29, 21, 15
TDBPP	4.75	697.6	360.4*, 441.2, 413.4	70	16, 13, 20

[a] Retention time

[b] Declustering potential

[c] Collision energy

[*] Quantitative ion

Table S14 Liquid chromatography gradient elution conditions [1].

Time (min)	A[a] (%)	B[b] (%)
0.01	25	75
2	20	80
6	5	95
10	5	95
13	25	75
15	5	95
15.01	Stop	

[a] 0.1% formic acid water;

[b] Methanol.

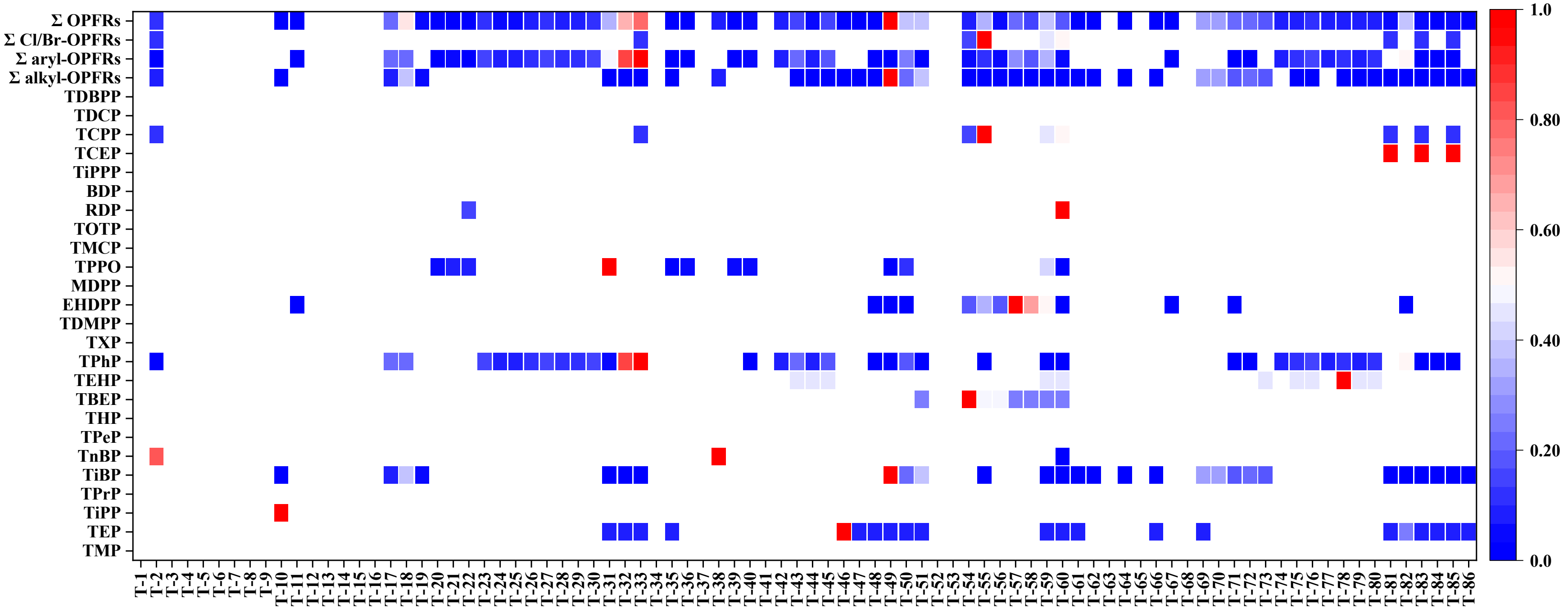

Figure S1 Heat map of OPFRs concentration in cosmetic sponges (normalized to maximum)

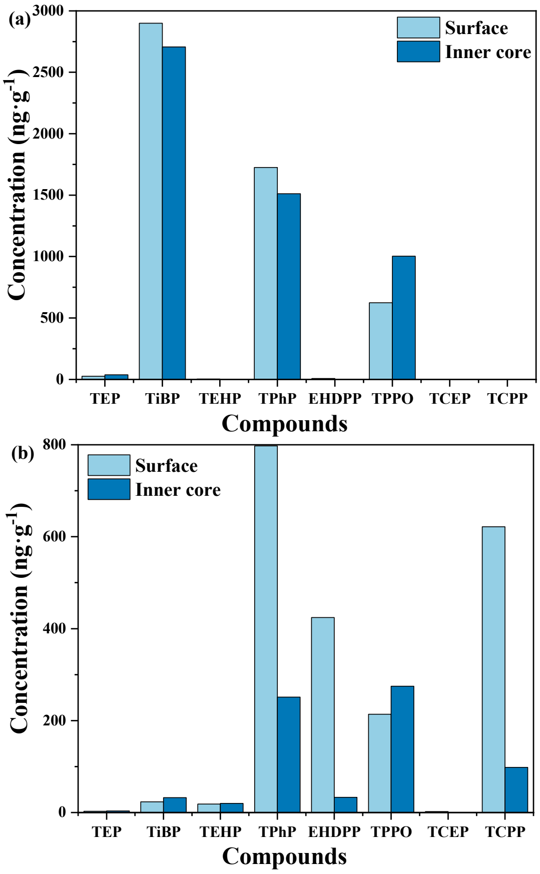

Figure S2 Mean concentrations of OPFRs on the surface and in the core of the sample (a) powder puffs (n=5); (b) beauty blenders (n=5)

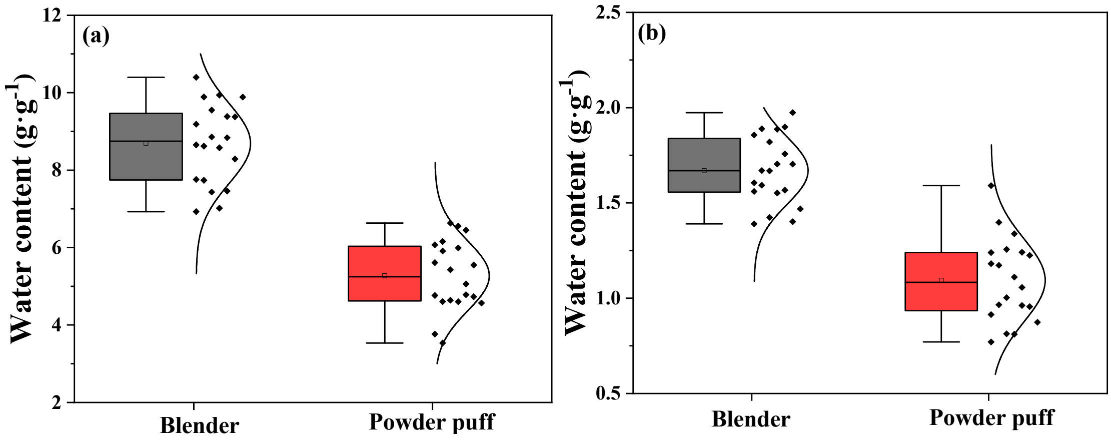

Figure S3 The water absorption capacity of cosmetic sponges for states of (a) water absorption saturation, and (b) water squeeze.

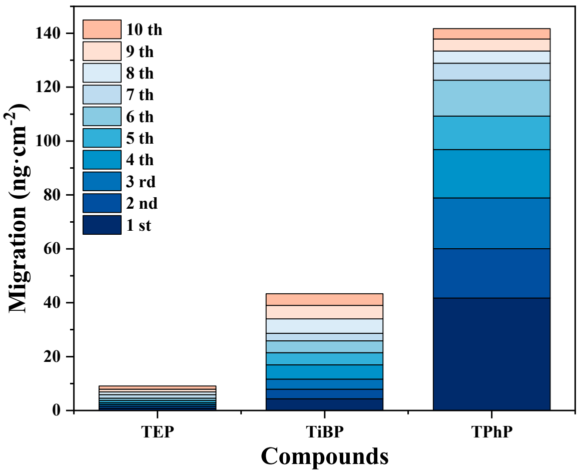

Figure S4 The migration of OPFRs for the wet cosmetic sponge (T-33) to be reused ten times

[1] Y. Yang, J. Ren, S. Tang, H. Cao, X.J. Zhou, J. Wang, Determination of 25 organophosphorus flame retardants in polyurethane products by dispersion solid phase extraction and liquid chromatography-tandem mass spectrometry, J. Chinese Mass Spectrom. Soc., 46 (2025), 206-216.
